# Supplementary material for: Mind Bomb Regulates Cell Death during TNF Signaling by Suppressing RIPK1’s Cytotoxic Potential
Source: Cell Rep. 2018 Apr 10;23(2):470–84. doi: 10.1016/j.celrep.2018.03.054 (PMC5912950; doi:10.1016/j.celrep.2018.03.054)
Supplement: Document S1. Supplemental Experimental Procedures and Figures S1–S7 [file mmc1.pdf]

Cell Reports, Volume *23*

Supplemental Information

Mind Bomb Regulates Cell Death

during TNF Signaling by Suppressing

RIPK1's Cytotoxic Potential

Rebecca Feltham, Kunzah Jamal, Tencho Tenev, Gianmaria Liccardi, Isabel Jaco, Celia Monteiro Domingues, Otto Morris, Sidonie Wicky John, Alessandro Annibaldi, Marcella Widya, Conor J. Kearney, Danielle Clancy, Paul R. Elliott, Timo Glatter, Qi Qiao, Andrew J. Thompson, Alexey Nesvizhskii, Alexander Schmidt, David Komander, Hao Wu, Seamus Martin, and Pascal Meier

Supplementary Information

Figure S1

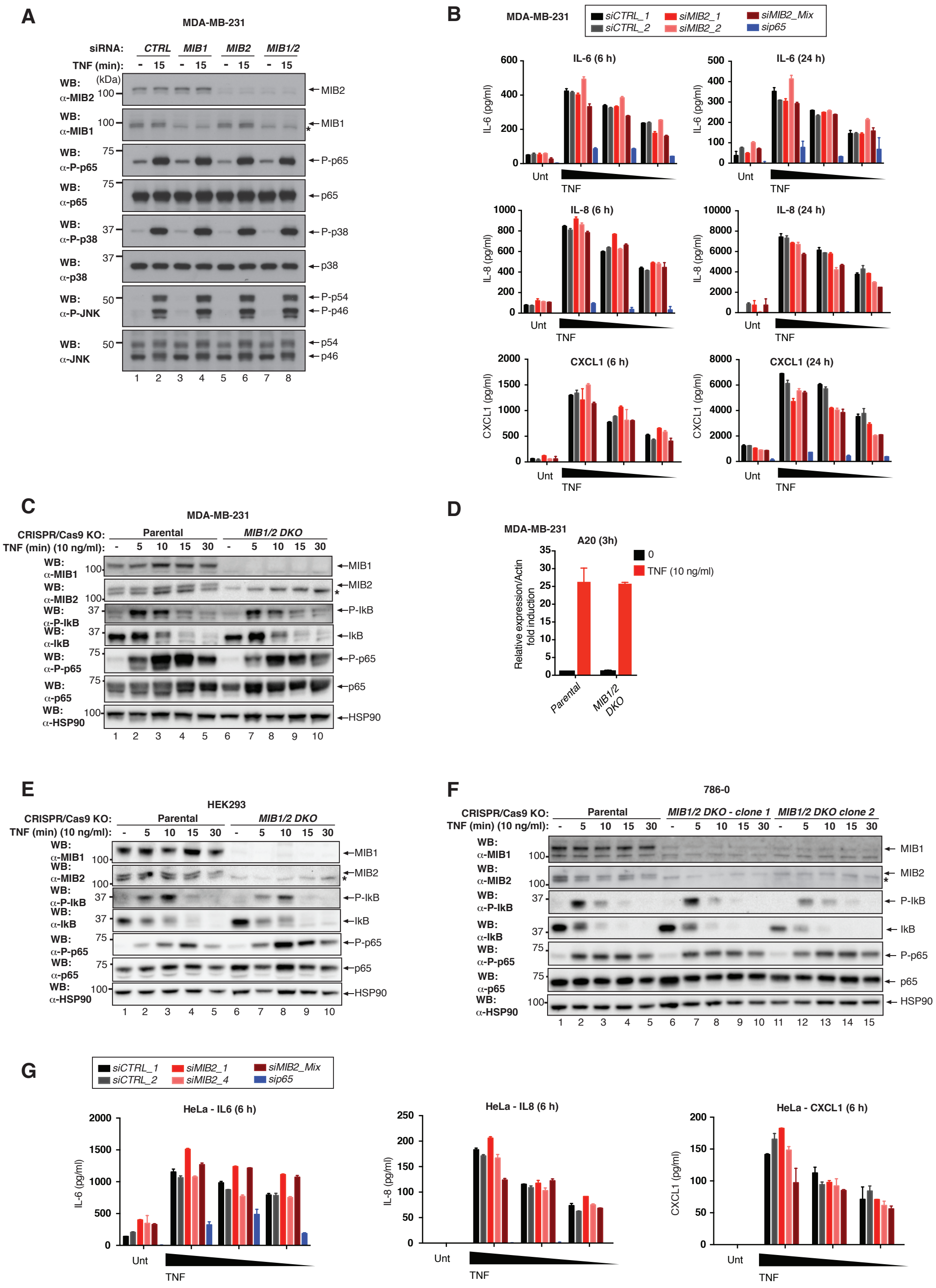

Supplementary Figure S1. MIB2 is not required for TNF-induced NF-ϰB activation and cytokine secretion, Related to Figure 1

(A) Western blot analysis of the indicated cell line subjected to RNAi-mediated knockdown of *MIB1*, *MIB2* or *MIB1*/*2*. Cells were either left untreated or treated with TNF (10 ng/ml) for 15 mins. (B) The presence of cytokines in the culture media of the indicated cell line was determined by ELISA. Cells were subjected to siRNA knockdown of *MIB2* or *p65* followed by stimulation with TNF at increasing concentrations (0.2-5 ng/ml) for 6 or 24 hrs. Error bars represent SD. (C) Comparison of TNF induced NF-ϰB activation in parental and *MIB1*/*2* DKO MDA-MB-231 cells. Cells were either left untreated or treated with TNF (10 ng/ml) for indicated times and lysates were analyzed by western blotting. (D) qRT-PCR analysis of mRNA from WT MDA-MB-231 and *MIB1*/*2* DKO MDA-MB-231 cells. Relative *A20* mRNA levels before and after 3 hrs of stimulation with 10 ng/ml TNF. (E-F) Comparison of TNF induced NF-ϰB activation in parental and *MIB1*/*2* DKO HEK293 cells (E) or 786-0 cells (F). Cells were either left untreated or treated with TNF (10 ng/ml) for indicated times and lysates were analyzed by western blotting. (G) The presence of cytokines in the culture media of HeLa cells was determined by ELISA. HeLa cells were subjected to siRNA-mediated knockdown of *MIB2* or *p65* followed by stimulation with TNF at increasing concentrations (0.2-5 ng/ml) for 6 or 24 hrs. Error bars represent SD.

Figure S2

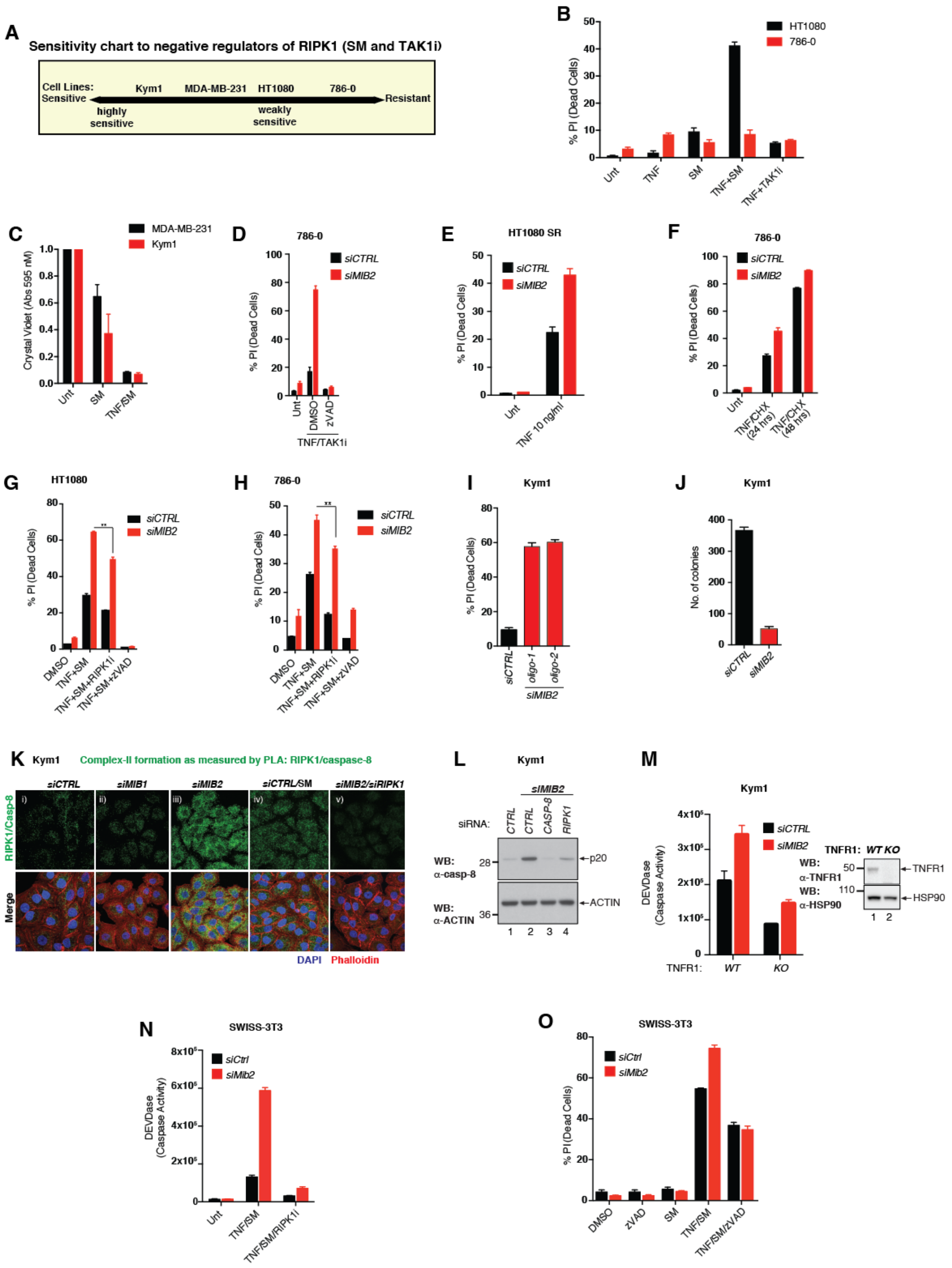

Supplementary Figure S2. MIB2 is required for TNF induced apoptosis, Related to Figure 2

(A) Schematic depicting the sensitivities of various cell lines to negative regulators of RIPK1. (B) FACS analysis of PI positive HT1080 and 786-0 cells treated with TNF (10 ng/ml), SM (100 nM), TNF/SM or TNF/TAK1i (1 μM) for 24 hrs. Error bars represent SD. (C) Crystal violet cell survival assay of Kym1 and MDA-MB-231 cells treated with SM (100 nM) or TNF (10 ng/ml)/SM (100 nM)

for 24 hrs. Error bars represent SD. (D) FACS analysis of PI positive 786-0 cells subjected to siRNA-mediated knockdown of *MIB2*. Following RNAi-mediated knockdown, cells were treated with DMSO or zVAD-FMK (10 μM) for 1 hr followed by treatment with TNF/TAK1i for 48 hrs. Error bars represent SD. (E) FACS analysis of PI positive HT1080 IkB^{SR} cells in which *MIB2* was knocked down by RNAi. Cells were treated with TNF (10ng/ml) for 24 hrs. Error bars represent SD. (F) FACS analysis of PI positive 786-0 cells subjected to siRNA-mediated knockdown of *MIB2*. Following RNAi-mediated knockdown, cells were treated with TNF (10 ng/ml) + CHX (10 μg/ml) for 24 or 48 hrs. Error bars represent SD. (G) Cell death analysis by Celigo of PI positive HT1080 cells subjected to siRNA-mediated knockdown of *MIB2*. Following RNAi-mediated knockdown, cells were treated with DMSO, zVAD-FMK (10 μM) or RIPK1i-GSK'963 (100 nM) for 1 hr followed by treatment with TNF/SM. Error bars represent SD. (H) Cell death analysis by Celigo of PI positive 786-0 cells subjected to siRNA-mediated knockdown of *MIB2*. Following RNAi-mediated knockdown, cells were treated with DMSO, zVAD-FMK (10 μM) or RIPK1i-GSK'963 (100 nM) for 1h followed by treatment with TNF/SM. Error bars represent SD. (I) FACS analysis of Kym1 cells treated with control siRNA oligos, or two independent oligos targeting *MIB2*. (J) Clonogenic growth assay using Kym1 cells subjected to siRNA knockdown of *MIB2*. 64 hrs post siRNA, 1000 cells were re-plated and left to form colonies. Error bars represent SEM. (K) Proximity ligation assay between RIPK1 and caspase-8 performed in Kym1 cells upon treatment with SM (100 nM) for 5 hrs or siRNA knockdown of *MIB1, MIB2, MIB2/RIPK1* for 96 hrs. All samples were treated with zVAD-FMK (10 μM) in fresh medium 16 hrs after transfection and then spiked with zVAD-FMK (10 μM) again at 48 hrs. (L) Western blot analysis of activated caspase-8 (p20 cleavage product) following siRNA knockdown of the indicated targets in Kym1 cells for 64 hrs. (M) DEVDase activity analysis of parental or *TNF-R1* KO Kym1 cells subjected to siRNA-mediated knockdown of *MIB2*. Following RNAi-mediated knockdown, cells were lysed and caspase activity was measured. (N) DEVDase assay using extracts from SWISS-3T3 cells subjected to siRNA knockdown of *Mib2*. Following RNAi-mediated knockdown, cells were treated with TNF/SM in presence or absence of RIPK1i-GSK'963 (100 nM) for 24 hrs. Error bars represent SD. (O) Cell death analysis by Celigo of PI positive SWISS-3T3 cells subjected to siRNA knockdown of *Mib2*. Following RNAi-mediated knockdown, cells were treated with the indicated agents for 17 hrs. Error bars represent SD.

Figure S3

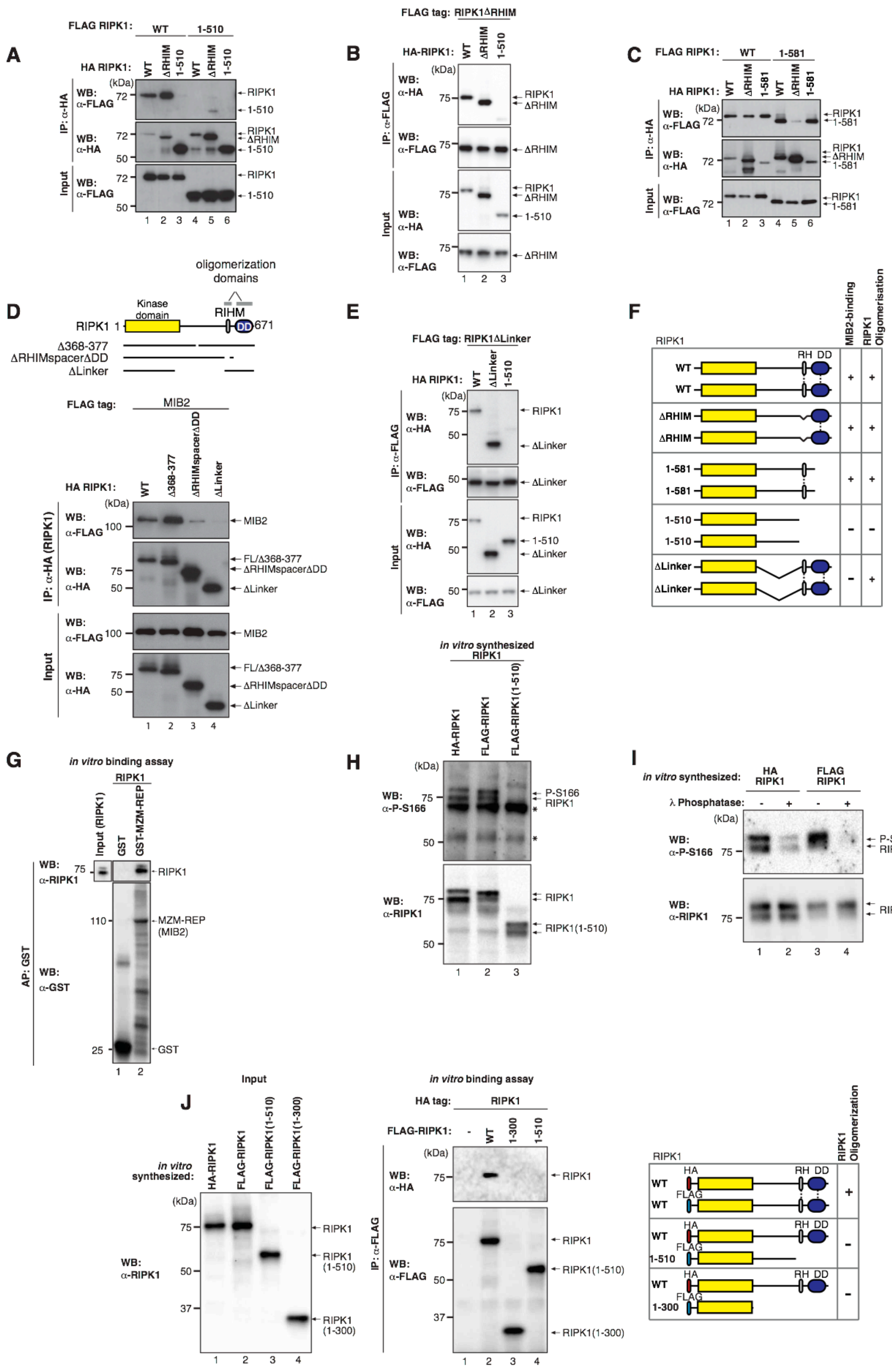

Supplementary Figure S3. MIB2 binds to oligomeric RIPK1, Related to **Figure 3**

(A-E) The indicated constructs were co-expressed in 293T cells. Immuno-precipitation was performed and interaction was assessed via western blot. (F) Schematic representation and summary of the results regarding MIB2 binding and RIPK1 homo-oligomerization. Indicated are the oligomerization and MIB2 binding capabilities of the various deletion constructs. (G) *In vitro* binding assay with

recombinant MIB2 and *in vitro* translated RIPK1. H) Western blot analysis of *in vitro* synthesized RIPK1 using the indicated antibodies. An asterisk indicates cross reactive bands. (I) Western blot analysis of *in vitro* synthesized RIPK1 treated with λ phosphatase as indicated. (J) *In vitro* binding assay of the indicated constructs. Left panel: input proteins. Middle panel: binding assay. FLAG-immuno-precipitation was performed and homo-oligomerization of *in vitro* synthesized RIPK1 proteins was assessed by Western blot. Right panel depicts the summary of the data.

Figure S4

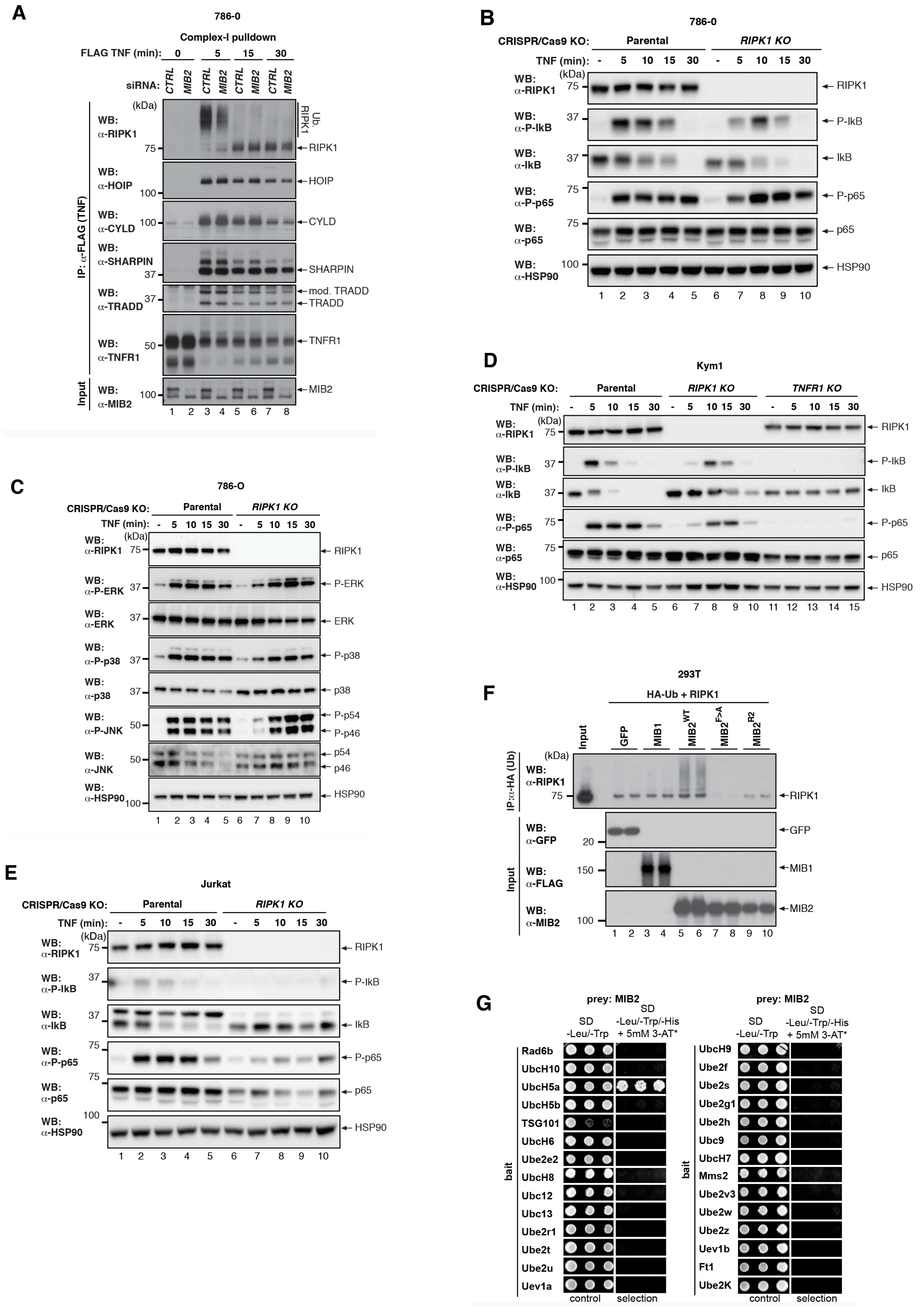

A
786-0
Complex-I pulldown
FLAG TNF (min): 0 5 15 30
siRNA: CTRL MIB2 CTRL MIB2 CTRL MIB2 CTRL MIB2
IP: α-FLAG (TNF)
Input
WB: α-RIPK1
WB: α-HOIP
WB: α-CYLD
WB: α-SHARPIN
WB: α-TRADD
WB: α-TNFR1
WB: α-MIB2
Ub. RIPK1
RIPK1
HOIP
CYLD
SHARPIN
mod. TRADD
TRADD
TNFR1
MIB2
B
786-0
CRISPR/Cas9 KO: Parental RIPK1 KO
TNF (min): - 5 10 15 30 - 5 10 15 30
WB: α-RIPK1
WB: α-P-IkB
WB: α-IkB
WB: α-P-p65
WB: α-p65
WB: α-HSP90
RIPK1
P-IkB
IkB
P-p65
p65
HSP90
C
786-O
CRISPR/Cas9 KO: Parental RIPK1 KO
TNF (min): - 5 10 15 30 - 5 10 15 30
WB: α-RIPK1
WB: α-P-ERK
WB: α-ERK
WB: α-P-p38
WB: α-p38
WB: α-P-JNK
WB: α-JNK
WB: α-HSP90
RIPK1
P-ERK
ERK
P-p38
p38
P-p54
P-p46
p54
p46
HSP90
D
Kym1
CRISPR/Cas9 KO: Parental RIPK1 KO TNFR1 KO
TNF (min): - 5 10 15 30 - 5 10 15 30 - 5 10 15 30
WB: α-RIPK1
WB: α-P-IkB
WB: α-IkB
WB: α-P-p65
WB: α-p65
WB: α-HSP90
RIPK1
P-IkB
IkB
P-p65
p65
HSP90
E
Jurkat
CRISPR/Cas9 KO: Parental RIPK1 KO
TNF (min): - 5 10 15 30 - 5 10 15 30
WB: α-RIPK1
WB: α-P-IkB
WB: α-IkB
WB: α-P-p65
WB: α-p65
WB: α-HSP90
RIPK1
P-IkB
IkB
P-p65
p65
HSP90
F
293T
HA-Ub + RIPK1
Input GFP MIB1 MIB2WT MIB2F>A MIB2R2
IP:α-HA (Ub)
Input
WB: α-RIPK1
WB: α-GFP
WB: α-FLAG
WB: α-MIB2
RIPK1
GFP
MIB1
MIB2
G
prey: MIB2
SD -Leu/-Trp
SD -Leu/-Trp/-His + 5mM 3-AT*
bait
Rad6b
UbcH10
UbcH5a
UbcH5b
TSG101
UbcH6
Ube2e2
UbcH8
Ubc12
Ubc13
Ube2r1
Ube2t
Ube2u
Uev1a
UbcH9
Ube2f
Ube2s
Ube2g1
Ube2h
Ubc9
UbcH7
Mms2
Ube2v3
Ube2w
Ube2z
Uev1b
Ft1
Ube2K
control
selection

Supplementary Figure S4. RIPK1 is a substrate for MIB2, Related to Figure 5

(A) 786-0 cells were subjected to siRNA of *MIB2* followed by TNF-induced complex-I immuno-precipitation. Cells were treated with FLAG-hTNF (0.8□μg/ml) for 0, 5, 15 and 30 mins followed by FLAG immuno-precipitation and western blot analysis. (B) Comparison of TNF induced NF-κB activation in parental and *RIPK1* KO 786-0 cells. Cells were either left untreated or treated with TNF (10 ng/ml) for indicated times and lysates were analyzed by western blotting. (C) Comparison of TNF-induced activation of ERK, p38 and JNK in parental and *RIPK1* KO 786-O cells. Cells were either left untreated or treated with TNF (10 ng/ml) for indicated times and lysates were analyzed by western blotting. (D) Comparison of TNF induced NF-κB activation in parental, *RIPK1* KO and *TNF-R1* KO Kym1 cells. Cells were either left untreated or treated with TNF (10 ng/ml) for indicated times and lysates were analyzed by western blotting. (E) Comparison of TNF induced NF-κB activation in parental and *RIPK1* KO Jurkat cells. Cells were either left untreated or treated with TNF (10 ng/ml) for indicated times and lysates were analyzed by western blotting. (F) Untagged GFP, MIB1, MIB2^{WT}, MIB2$^{F>A}$ or MIB2^{R2} was co-expressed with HA-Ub and untagged RIPK1 in 293T cells. HA-immuno-precipitation was performed and ubiquitylation of RIPK1 was assessed via western blot. (G) Yeast-two-hybrid assay screening the interaction of the RING finger of MIB2 (encoding amino acids 843-1000) with 22 human E2s and 6 human Ub-conjugating E2 variants (TSG101, Uev1a, Uev1b, Mms2, Ube2v3, Ft1).

Figure S5

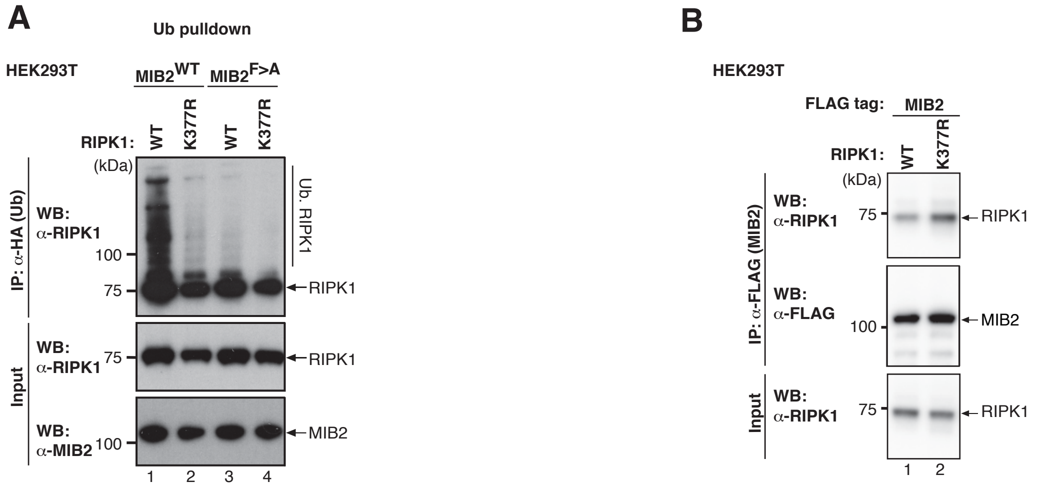

Supplementary Figure S5. RIPK1K377R mutation does not abrogate binding to MIB2, Related to Figure 6

(A) RIPK^{WT} and RIPK1^{K377R} were co-expressed with the indicated constructs in 293T cells. HA-immuno-precipitation was performed and ubiquitylation of RIPK1 was assessed via western blot. (B) FLAG MIB2 was co-expressed with untagged RIPK1^{WT} or RIPK1^{K377R} in 293T cells. FLAG-immuno-precipitation was performed and interaction with RIPK1 was assessed via western blot.

Figure S6

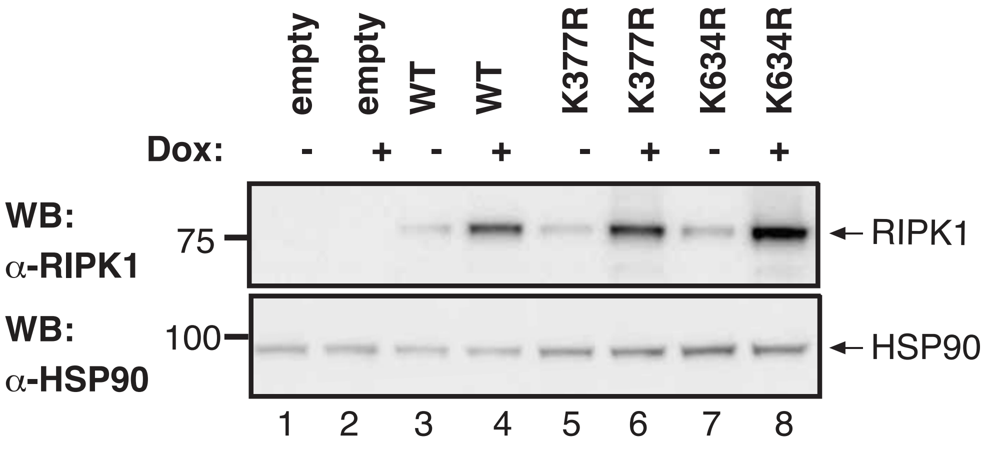

Supplementary Figure S6. Expression of RIPK1 mutants in MDA-MB-231 cells, Related to Figure 6

Western blot analysis of lysates from parental and *RIPK1* KO MDA-MB-231 cells reconstituted with either RIPK1^{WT}, RIPK1^{K377R} and RIPK1^{K377R} were induced with doxycycline for 6 hrs.

Figure S7

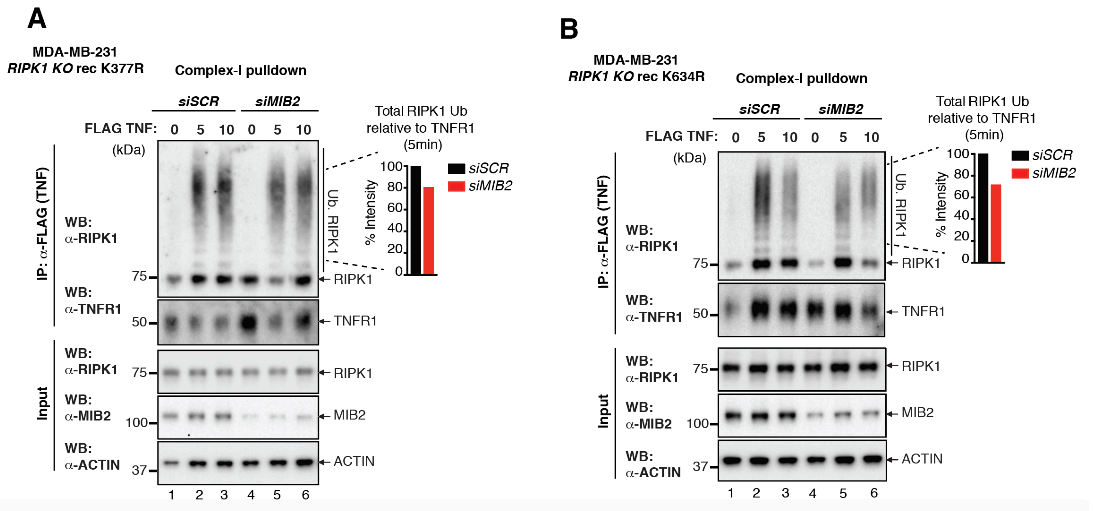

Supplementary Figure S7. MIB2 ubiquitylates RIPK1 at multiple lysine residues, Related to Figure 7

(A-B) Ubiquitylation of RIPK1 in complex-I. *RIPK1* KO MDA-MB-231 cells were reconstituted with either *RIPK1*^{WT} or *RIPK1*^{K377R} (A) or *RIPK1*^{K634R} (B). Cells were subjected to RNAi-mediated knockdown of *MIB2*. 48 hrs later cells were treated with FLAG-hTNF (0.8 μg/ml) for the indicated time-points post 5 hrs doxocycline induction, followed by FLAG immune-precipitation and western blot analysis.

Supplemental Experimental Procedures

Resource Table

REAGENT or RESOURCE	SOURCE	IDENTIFIER
Antibodies		
α-RIPK1 (N-terminal)	Cell Signaling	Cat#3493
α-HA	Roche	Cat#11867423001
α-MIB2	Bethyl Laboratories	Cat#A301-414A
α-MIB1	Gift from Patricia J. Gallagher	N/A
α-CYLD	Cell Signaling	Cat#8462; D1A10
α -SHARPIN	Proteintech	Cat#14626-1-AP
α-HOIL	Gift from Henning Walczak	N/A
α-HOIP	Bethyl Laboratories	Cat#A303-560A
α-NEMO	Santa Cruz Biotechnology	Cat#sc-8330
α-TRADD	BD Biosciences	Cat#610572
α-cIAP1	Enzo Life Sciences	Cat#ALX-803-335-C100
α-TAK1	Cell Signaling	Cat#4505
α-TNF-R1	Abcam	Cat#19139
α-TNF-R1	Santa Cruz Biotechnology	Cat#sc-8436
α-ACTIN	Sigma	Cat#A5441
α-P-p65	Cell Signaling	Cat#3033
α-p65	Cell Signaling	Cat#8242
α-IκBα	Santa Cruz Biotechnology	Cat#sc-371
α-P-IκBα	Cell Signaling	Cat#2859
α-P-p38	Cell Signaling	Cat#9215
α-p38	Cell Signaling	Cat#9212
α-P-JNK	Cell Signaling	Cat#4668
α-JNK	Santa Cruz Biotechnology	Cat#sc-571
α-P-ERK	Cell Signaling	Cat#9101
α-CASPASE-8 - for WB - post IP	MBL	Cat#M032-3
α-CASPASE-8 - for IP [C-20]	Santa Cruz Biotechnology	Cat#sc-6136
α-CASPASE-8 for cleavage	R&D	Cat#AF1650
α-FLAG [M2]	Sigma	Cat#F3165
α-Ub	Dako	Cat#Z0458
α-PARP1 [F2]	Santa Cruz Biotechnology	Cat#sc-8007
α-FLIP	Enzo Life sciences	Cat#ALX-804-428-C050
α-GFP	Santa Cruz Biotechnology	Cat#sc-8334
α-MYC	Sigma	Cat#M5546
α-HSP90	Santa Cruz Biotechnology	Cat#sc-7947
α-A20	Cell Signaling	Cat#5630
α-TRAF2	Santa Cruz Biotechnology	Cat#sc-876
α-ERK	Gift from Chris Marshall	N/A
α-pERK	Sigma	M8159
Chemicals, Peptides, and Recombinant Proteins		
Enbrel	Wyeth	N/A
Biotin-AHX-Ub-PA	(UbiQ)	Cat#UbiQ-076
FLAG-hTNF	Enzo Life Sciences	Cat#ALX-804-034-C050

FLAG-hTNF	Gift from Henning Walczak	N/A
(5Z)-7-Oxozeaenol (TAK1 inhibitor)	Tocris	Cat#3604
zVAD-FMK	Apex Bio	Cat#A1902
QVD	Apex Bio	Cat#A1901
SM-164	Gift from Shaomeng Wang	N/A
LPS	Invivogen	Cat#TLRL-PEKLPS
Ac-DEVD-AMC	Cambridge Bio	Cat#CAY14986
Hoechst	Thermo Scientific	Cat#33342
Propidium iodide solution (PI)	Sigma	Cat#P4864
MTT reagent	Sigma	Cat#M5655
Protein A/G agarose	Thermo Scientific	20423
Halt Protease and phosphatase inhibitor	Thermo Scientific	78443
PR619	2B Scientific	SI9619
GSK'963 (RIPK1 inhibitor)	Gift from GSK	N/A
Human TaqMan A20 Probe Hs00234713_m1	Thermo Scientific	Cat#4331182
Human TaqMan ACTIN Probe Hs01060665_g1	Thermo Scientific	Cat#4331182
Critical Commercial Assays		
RNAEasy	Qiagen	Cat#74106
QuantiTech reverse transcription	Qiagen	Cat#205314
Duolink In Situ Detection Reagents Green	Sigma	DUO92014
Experimental Models: Cell Lines		
HT1080$^{IkB\text{-}SR}$	Gift from O. Micheau (Dijon, France)	N/A
Kym1	Gift from John Silke (Melbourne, Australia)	N/A
HT1080	ATCC	Cat#CCL-121
MDA-MB-231	In house	N/A
HEK293T	In house	N/A
Flp-In^{TM}T-REx^{TM}-HEK293	Termo Scientific	Cat#R78007
786-0	In house	N/A
SWISS-3T3	In house	N/A
Oligonucleotides		
shRNA Mib2	Thermo Scientific	Clone ID: V3THS_324301
siMIB2_1 [Hs_ZZANK1_4 (hMib2)]	Qiagen	Cat#SI00779688
siMIB2_4 [hs_Mib2_4]	Qiagen	Cat#SI04369778
siMIB2_6 [hs_Mib2_6]	Qiagen	Cat#SI05126436
siALL*Control	Qiagen	Cat#1027281
Recombinant DNA		
Cas9-plasmid	Addgene	Cat#41815 or 48138
Mib2 cDNA	Gift from Vanessa Redecke	N/A
pcDNA3	Thermo Scientific	Cat#V79020
Deposited Data		
http://dx.doi.org/10.17632/52t6f2m8k5.1		
Software and Algorithms		

CRISPR design	http://crispr.mit.edu	(Ran et al., 2013)
CRISPR design	http://www.addgene.org/crispr/church/	(Mali et al., 2013)
SAINT analysis	http://saint-apms.sourceforge.net/	(Choi et al., 2011)
Swiss-Prot	https://www.ebi.ac.uk/uniprot	
Proteome Discoverer v1.4	Thermo Scientific	Cat#IQLAAEGABSFAKJMAUH
Image Lab V5.2.1.	Bio-Rad laboratories	
Sequence alignment	http://benchling.com	
GraphPad Prism v6.0	http://www.graphpad.com/	

CONTACT FOR REAGENTS AND RESOURCE SHARING

Further information and requests for reagents may be directed to Pascal Meier (pmeier@icr.ac.uk).

Plasmids

The *MIB2* cDNA (kind gift from Vanessa Redecke) was altered by mutagenesis to correspond to Q96AX9-2 - MIB2_human (UniProt). All constructs used for transient transfection experiments were cloned into pcDNA3 mammalian expression vector (Invitrogen) and sequence verified. For generation of stable cell lines Lentiviral tet-On inducible vector pTRB3A1 was used and cells were selected in the presence of Blasticidin.

RNA Interference, Transfections and Infections

Unless otherwise indicated, all siRNA assays were performed using a total of 50 nM – 100 nM of siRNA. When multiple siRNAs were combined, each siRNA was used at 25 nM and control siRNA was used where required to balance siRNA concentrations so equal amounts were transfected. All siRNA transfections unless otherwise stated were performed using DharmaFECT4 transfection reagent (GE Healthcare) and Opti-MEM (Life Technologies). All siRNA transfections unless otherwise stated were performed using retro transfection and left for 40 hrs from the time of transfection to facilitate knockdown. Unless otherwise indicated siMIB2 refers to the combination of hs_ZZANK1_4 (25 nM) + hs_MIB2_2 (25 nM) + hs_MIB2_4 (25 nM) + siCtrl (25 nM). For experiments where *siMIB1* and *siMIB2* are co-knocked down, *siMIB2* refers to the combination of hs_MIB2_2 (25 nM) + hs_MIB2_4 (25 nM) + *siCtrl* (50 nM) or *siMIB1* (50 nM). For all ELISA experiments $5x10^5$ cells were electroporated with 10 µl of 20 µM siRNA then seeded at $3x10^4$ in 24-well plates for 48 hrs. Generation of lentiviral particles was conducted as described previously (Vince et al., 2008; Vince et al., 2007).

Caspase-8 Cleavage Assays

Cells were seeded in 6-well plates and treated as indicated. Cells were lysed in 200 µl of DISC lysis buffer supplemented with 2% SDS, protease and phosphatase inhibitors. Cell lysates were passed through 0.8 ml columns (Pierce) to shred genomic DNA. Proteins lysates were quantified before separating samples by SDS-PAGE using NuPAGE Novex 4-12% Bis-Tris 1.0 mm 12 well precast

protein gels (Invitrogen) in MES buffer. Caspase-8 cleavage antibody [AF1650] (R&D) was used to detect cleavage products.

Cell Death (FACS) and MTT assays

Cells were plated in 96-well plates and retro siRNA transfection was performed for 40 hrs. Cells were treated as indicated in 150 µl for indicated times. Medium containing dead cells was transferred to a round bottom 96 well plate, live cells were trypsinized in 50 µl, live cells were harvested with 100 µl of medium containing 1 µg/ml PI with 2.5 mM $CaCl_2$ and 2.5 µl/ml AnnexinV antibody (BD Biosciences) and combined with dead cells (total volume 300 µl). 96-well plate was analyzed by FACS using a plate reader. Data shown are from 5000 cells per condition. For CeligoS assays, 5 x10^4 786-O or HT1080 cells were seeded in 96-well plates and 24 h later cells were treated as indicated for the indicated times. Hoechst (0.5 µg/ml) and PI (1 µg/ml) were added and the percentage of dead cells was measured using the CeligoS image cytometer (Nexcelon Bioscience). For MTT assays 5x10^4 cells were seeded in 24-well plates. Cells were treated as indicated in 500 µl for indicated times, after which 50 µl of MTT reagent re-suspended in H_20 to a concentration of 5 mg/ml was added to the cells and left to develop for 2 hrs. Media was removed and 500 µl DMSO added to solubilize the crystals. Absorbance was read on a spectrometer at 570 nm.

Crystal Violet Cell Survival Assays

Cells were seeded in a 6-well plate. The following day the indicated treatments were added in 2 ml of DMEM and the cells were left for 24 hrs in treated medium. Cells were fixed in 3.7% formaldehyde/PBS for 10 mins and stained with crystal violet/PBS for 10 mins. Colonies were either dissolved in 1 ml of 10% acetic acid and the absorbance read on a spectrometer at 595 nm.

Clonogenic Assays

Cells were seeded in a 6-well plate and retro siRNA transfection was performed. After 40 hrs (786-0) or 64 hrs (Kym1) cells were trypsinized, counted and 1000 viable cells were re-plated into a 6-well plate. The following day the indicated treatments were added in 2 ml of DMEM and the cells were left for approximately 9 days in treated medium. Cells were fixed in 3.7 % formaldehyde/PBS for 10 mins and stained with crystal violet/PBS for 10 mins. Colonies were either dissolved in 1 ml of 10% acetic acid and the absorbance read on a spectrometer at 595 nm, or the colonies were counted and recorded using Image J.

Caspase activity assays (DEVDase)

DEVDase assay was performed as previously described (Jaco et al., 2017). In brief, cells were plated in 96-well plates and retro siRNA transfection was performed for 40 hrs. After treatment, medium was removed and 1 % DISC lysis buffer (20 mM Tris-HCL pH7.5, 150 mM NaCl, 2 mM EDTA, 1 % Triton X-100, 10 % Glycerol, H_20) was added to each well. Plates were placed at -80 °C to aid cell lysis. Plates were thawed at room temperature for 15 mins, after which DEVDase assay mix was added to each well (NB: cell lysates were not cleared). The plates were wrapped in foil and the reaction was

incubated at room temperature for up to 24 hrs. DEVDase activity was read at 380 nM excitation/460 nM emission.

TUBE Assays

Cells were lysed in DISC lysis buffer (20 mM Tris-HCL pH7.5, 150 mM NaCl, 2mM EDTA, 1% Triton X-100, 10% Glycerol, H_20) supplemented with protease inhibitors, 1 mM DTT, PR619 (10 µM), GST-TUBE (50 µg/ml; 50 µg TUBE/mg protein lysate). Cell lysates were rotated at 4 °C for 20 mins then clarified at 4 °C at 14,000 rpm for 10 mins. 20 µl GST beads were added and immuno-precipitation was performed overnight. 4x washes in wash buffer (50 mM Tris pH 7.5, 150 mM NaCl, 0.1 % Triton X-100, and 5 % glycerol) + PR619 (10 µM) were performed, and samples eluted by boiling in 50 µl 1x SDS loading dye.

Homology Modeling

A homology model of human RIPK1 Death Domain (DD, 583-669) was generated by the SWISS-MODEL server using PIDD DD structure as the template (PDB code 2OF5). Then modeled RIPK1 DD structure was aligned to FAS DD in FAS/FADD complex structure (PDB code 3OQ9) to form a RIPK1 DD/FADD DD complex structure. The alignment was performed by Coot and figures were made by PyMol.

Complex-I/II Purification

Complex-I/II purification was essentially performed as previously described (Jaco et al., 2017). In brief, cells were seeded in 15 cm dishes and treated as indicated using pre-warmed media containing 3xFLAG-hTNF (0.8 µg/ml). After stimulation media was removed and plates were washed with ice cold PBS to stop stimulation and frozen at -80 °C. Plates were thawed and cells were lysed in DISC lysis buffer supplemented with protease inhibitors and PR619 (10 µM). Cell lysates were rotated at 4 °C for 20 mins then clarified at 4 °C at 14,000 rpm for 10 mins. 20 µl of anti-FLAG M2 beads (SIGMA) were rotated with cleared protein lysates overnight at 4 °C. 0 hr sample: 0.8 µg/ml of FLAG-TNF was added post-lysis. 4x washes in DISC buffer with PR619 (10 µM) were performed, and samples eluted by boiling in 50 µl 1x SDS loading dye. For complex-II purification cells were seeded in 10 cm dishes and treated as indicated in figure legends. Cells were lysed on ice as above. Cell lysates were rotated at 4 °C for 20 mins then clarified at 4 °C at 14,000 rpm for 10 mins. 20 µl of protein G sepharose (SIGMA) with Caspase-8 (C20) antibody (Santa Cruz Biotechnology) (1.5 µg antibody/mg protein lysate) were rotated with cleared protein lysates overnight at 4 °C. 4x washes in wash buffer (50 mM Tris pH 7.5, 150 mM NaCl, 0.1% Triton X-100, and 5% glycerol) were performed, and samples eluted by boiling in 50 µl 1x SDS loading dye.

Ubiquitylation Assays

0.4 µg of plasmids expressing HA-Ub or Myc-Ub was transfected into 293T cells in combination with the indicated constructs. Transfection was left for 16 hrs after which cells were lysed with DISC lysis buffer supplemented with protease inhibitors and PR619 (10 µM). Cell lysates were rotated at 4 °C for

20 mins then clarified at 4°C at 14,000 rpm for 10 mins. 20 µl anti-HA beads (SIGMA) or 20 µl of protein G sepharose (SIGMA) + 3 µl anti-Myc were rotated with cleared protein lysates overnight at 4 °C. 4x washes in wash buffer (50 mM Tris pH 7.5, 150 mM NaCl, 0.1% Triton X-100, and 5% glycerol) supplemented with PR619 (10 µM) were performed, and samples eluted by boiling in 50 µl 1x SDS loading dye.

In vitro Binding and Ubiquitylation Assay

All constructs for *in vitro* translation assays were cloned into pcDNA3 and translated using the Promega TNT Coupled Reticulocyte Lysate System. For production of GST-tagged proteins, MIB2 constructs were expressed from pGEX6p-1 in BL21(DE3)/pLysS strain and purified with GST beads. All *in vitro* binding assays were conducted in the presence of DISC buffer overnight at 4 °C. Beads were washed 4 times with the same buffer and protein complexes were eluted by boiling the beads in 1x SDS loading dye. For the *in vitro* assay shown in Fig 5G, purified Strep-tagged MIB2 was incubated with recombinant GST-tagged full length RIPK1 (Abnova), E1 enzyme preloaded with Ub (Boston Biochem), UbcH5a (Boston Biochem) and ATP in Ub assay buffer (40 mM Tris-HCL pH 7.5, 10 mM MgCl2, 0.6 mM DTT) at 37 °C for 90 mins. Reactions were stopped by adding SDS loading dye and samples were analysed by Western blot with the indicated antibodies.

UbiCRest

The UbiCRest analysis with linkage selective DUBs was performed essentially as previously described (Hospenthal et al., 2015). Briefly, the release fraction (see above) was incubated with the following DUBs: 1 µM OTULIN, 0.2 µM OTUD1, 1 µM CEZANNE, 0.2 µM OTUB1, 1.5 µM USP21, 0.5 µM vOTU. The reaction was conducted in the presence of 1 mM DTT for 30 min at 37 °C. Reactions were stopped with loading buffer, and the ubiquitylation status analyzed by western blotting.

Proximity Ligation Assay

PLA was performed according to the manufacturer’s protocol using the Duolink Detection Kit (SIGMA). Cells were examined with a confocal microscope (objective x 40, Zeiss LSM 710).

Directed Yeast Two-Hybrid Assays

The yeast strain Y2HGold (Clontech) was co-transformed with pGBT9-MIB2 (encoding amino acids 843-1000) as a bait and the respective prey plasmids encoding 22 human E2s and 6 human Ub-conjugating E2 variants. Positive transformants were selected on minimal SD-Leu-Trp medium (Formedium). Three single colonies for each bait and prey co-transformation were patched out on fresh SD-Leu-Trp plates and grown for 2 days at 30 °C. Each patch was re-suspended in 180 µl of sterile water in a 96 well plate and plated in replicate onto non-selective (SD-Leu-Trp) or selective medium (SD-Leu-Trp-His, containing 5 mM of 3-amino-1,2,4-triazole (3-AT, Formedium)). Yeasts were incubated at 30ºC for 1 week. The E2s/UEVs library was kindly provided by Rachel Klevit.

qRT-PCR

qRT-PCR was performed as previously described (Morris et al., 2016), with some modifications. MDA-MB-231 parental and *MIB1/2 DKO* were treated with TNF (10 ng/ml) for 3 hrs and immediately frozen. qRT-PCR was performed using Taqman gene expression mastermix (Thermo Fisher Scientific) and the QuantStudio 6 Flex Real-Time PCR System. The amount of mRNA detected was normalized to control *ACTIN* mRNA values. The relative ΔCt sample/ΔCtr Actin ratios of WT controls were set at 100%, and the fold differences were calculated using the ΔΔCt method.

Mass Spectrometry

Prior to mass spectrometry analysis of RIPK1 interactors, eluted protein complexes were digested with Trypsin and peptides were purified using C18 Microspin columns (Harvard Apparatus) according to the manufactures instruction. LC-MS/MS analysis was performed on a dual pressure LTQ-Orbitrap mass spectrometer (Thermo Scientific), which was connected to an electrospray ion source (Thermo Scientific). Peptide separation was carried out using an easy nano-LC systems (Proxeon Biosystems) equipped with an RP-HPLC column packed with C18 resin (Magic C18 AQ 3 μm; Michrom BioResources). A 0.3 μl/min linear gradient from 96 % solvent A (0.15 % formic acid, 2 % acetonitrile) and 4 % solvent B (98 % acetonitrile, 0.15 % formic acid) to 40 % solvent B over 40 min. The data acquisition mode was set to obtain one high-resolution MS scan in the FT part of the mass spectrometer at a resolution of 60,000 FWHM followed by MS/MS scans in the linear ion trap of the 20 most intense ions. Raw files were converted to the mzXML format, and searched against the human swissprot protein database. Further data processing including SAINT was carried out as described previously (Choi et al., 2011). For the identification of ubiquitylated sites on RIPK1 by MIB2, 293T cells were transfected with 3xHA-RIPK1, MIB2 and Ub or 3xHA-RIPK1, MIB2F>A and Ub for 48 hrs. After lysis with DISC lysis buffer in the presence of protease inhibitors and PR619, HA affinity purification was performed. Bound complexes were eluted with 5 % formic acid and then submitted for analysis by LC-MS/MS using a tryptic digestion workflow. Specific accurate mass (+/- 10 ppm) and retention time (AMRT) profiles were determined for the peptides of interest, accounting for the following variable modification states: unmodified and diGly-modification of lysine. Additionally, the sum value of four RIPK1 peptides was calculated relative to the data from the RIPK1 sample. The four peptides were selected as proxies to indicate the level of RIPK1 in the two samples. LC-MS/MS analysis of ubiquitylated peptides was performed after immuno-precipitation. Elutes were dried in vacuo and reconstituted in 50 mM triethylammonium bicarbonate. Samples were then reduced with 5 mM tris(2-carboxyethyl)phosphine, free cysteines were alkylated with 10 mM chloroacetamide or chloroacetic acid and protein was digested with trypsin. For the targeted analysis of residue K377, a second digestion was performed using endoproteinase Glu-C. The resulting peptides were analysed by direct injection on an Agilent 1200 nanoLC (Agilent Technologies) in-line with an LTQ Velos Orbitrap mass spectrometer (Thermofisher Scientific) with the following modifications: Peptides were resolved over 30 mins using a linear gradient of 96:4 to 50:50 buffer A:B (buffer A: 1% acetonitrile/3 % dimethyl sulfoxide/0.1 % formic acid; buffer B: 80 % acetonitrile/3 % dimethyl sulfoxide/0.1 % formic acid) at 250 nL/min. The ion at 401.922718 m/z was used for FT-MS internal lock mass calibration. Peak lists were extracted using Proteome Discoverer v1.4 and interrogated using Mascot v2.3 against

the Swissprot 2015_04 Homo Sapiens subset database (20,273 sequences) customized to include construct sequences as required. Residual Ub signatures GG and LRGG were included as variable modifications at lysine residues.

Supplemental References

Choi, H., Larsen, B., Lin, Z.Y., Breitkreutz, A., Mellacheruvu, D., Fermin, D., Qin, Z.S., Tyers, M., Gingras, A.C., and Nesvizhskii, A.I. (2011). SAINT: probabilistic scoring of affinity purification-mass spectrometry data. Nat Methods *8*, 70-73.

Hospenthal, M.K., Mevissen, T.E., and Komander, D. (2015). Deubiquitinase-based analysis of ubiquitin chain architecture using Ubiquitin Chain Restriction (UbiCRest). Nature protocols *10*, 349-361.

Jaco, I., Annibaldi, A., Lalaoui, N., Wilson, R., Tenev, T., Laurien, L., Kim, C., Jamal, K., Wicky John, S., Liccardi, G., *et al*. (2017). MK2 Phosphorylates RIPK1 to Prevent TNF-Induced Cell Death. Mol Cell *66*, 698-710 e695.

Mali, P., Yang, L., Esvelt, K.M., Aach, J., Guell, M., DiCarlo, J.E., Norville, J.E., and Church, G.M. (2013). RNA-guided human genome engineering via Cas9. Science *339*, 823-826.

Morris, O., Liu, X., Domingues, C., Runchel, C., Chai, A., Basith, S., Tenev, T., Chen, H., Choi, S., Pennetta, G., *et al*. (2016). Signal Integration by the IkappaB Protein Pickle Shapes Drosophila Innate Host Defense. Cell Host Microbe *20*, 283-295.

Vince, J.E., Chau, D., Callus, B., Wong, W.W., Hawkins, C.J., Schneider, P., McKinlay, M., Benetatos, C.A., Condon, S.M., Chunduru, S.K., *et al*. (2008). TWEAK-FN14 signaling induces lysosomal degradation of a cIAP1-TRAF2 complex to sensitize tumor cells to TNFalpha. J Cell Biol *182*, 171-184.

Vince, J.E., Wong, W.W., Khan, N., Feltham, R., Chau, D., Ahmed, A.U., Benetatos, C.A., Chunduru, S.K., Condon, S.M., McKinlay, M., *et al*. (2007). IAP antagonists target cIAP1 to induce TNFalpha-dependent apoptosis. Cell *131*, 682-693.
