# Supplementary material for: Mind Bomb Regulates Cell Death during TNF Signaling by Suppressing RIPK1’s Cytotoxic Potential
Source: Cell Rep. 2018 Apr 10;23(2):470–84. doi: 10.1016/j.celrep.2018.03.054 (PMC5912950; doi:10.1016/j.celrep.2018.03.054)
Supplement: Document S2. Article plus Supplemental Information [file mmc2.pdf]

Cell Reports

Article

Mind Bomb Regulates Cell Death during TNF Signaling by Suppressing RIPK1's Cytotoxic Potential

Graphical Abstract

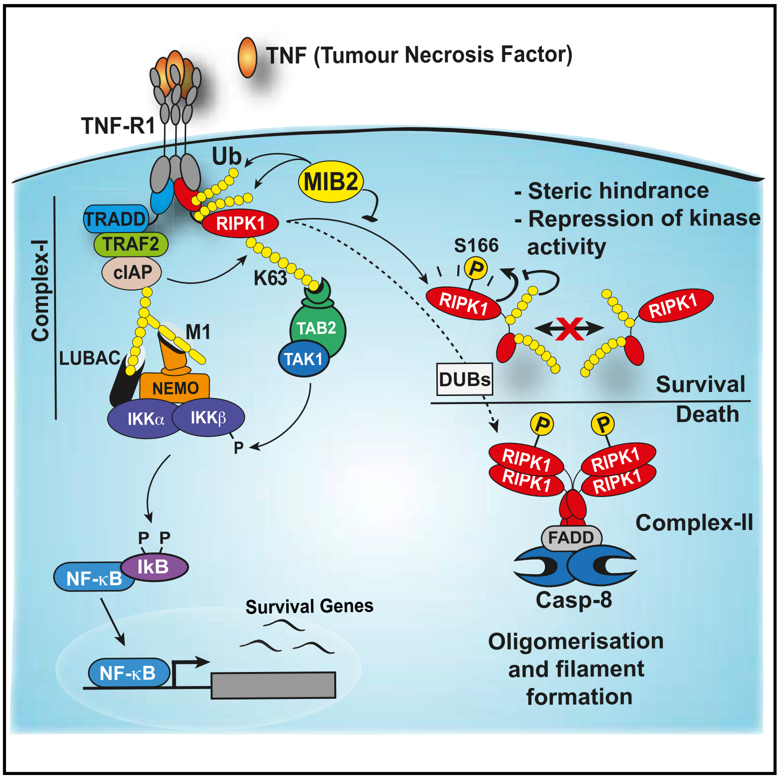

Highlights

- RIPK1 recruits the E3 ligase MIB2 to the TNF receptor signaling complex-I
- Depletion of MIB2 sensitizes cells to RIPK1- and caspase-8-dependent cell death
- MIB2-mediated ubiquitylation of RIPK1 represses its kinase activity and cell death
- MIB2 acts as an additional survival checkpoint in TNF signaling

Authors

Rebecca Feltham, Kunzah Jamal, Tencho Tenev, ..., Hao Wu, Seamus Martin, Pascal Meier

Correspondence

pmeier@icr.ac.uk

In Brief

Feltham et al. show that MIB2 directly ubiquitylates RIPK1 upon TNF stimulation, suppressing the cytotoxic potential of RIPK1 and acting as a checkpoint within the TNF signaling pathway.

Feltham et al., 2018, Cell Reports *23*, 470–484
April 10, 2018 © 2018 The Author(s).
https://doi.org/10.1016/j.celrep.2018.03.054

CellPress

OPEN ACCESS
CellPress

Cell Reports
Article

Mind Bomb Regulates Cell Death during TNF Signaling by Suppressing RIPK1's Cytotoxic Potential

Rebecca Feltham,[1,9,10] Kunzah Jamal,[1,10] Tencho Tenev,[1,10] Gianmaria Liccardi,[1] Isabel Jaco,[1,2] Celia Monteiro Domingues,[1] Otto Morris,[1] Sidonie Wicky John,[1] Alessandro Annibaldi,[1] Marcella Widya,[1] Conor J. Kearney,[3] Danielle Clancy,[3] Paul R. Elliott,[4] Timo Glatter,[5,6] Qi Qiao,[7] Andrew J. Thompson,[1] Alexey Nesvizhskii,[8] Alexander Schmidt,[5] David Komander,[4] Hao Wu,[7] Seamus Martin,[3] and Pascal Meier[1,11,*]

[1]The Breast Cancer Now Toby Robins Research Centre, Institute of Cancer Research, Fulham Road, London SW3 6JB, UK
[2]AstraZeneca, IMED Oncology, Bioscience, DDR Group, Chesterford Research Park, Little Chesterford CB10 1XL, UK
[3]Molecular Cell Biology Laboratory, Department of Genetics & The Smurfit Institute, Immunology Research Centre, Trinity College, Dublin 2, Ireland
[4]Medical Research Council, Laboratory of Molecular Biology, Cambridge, UK
[5]Proteomics Core Facility, Biocentrum of the University of Basel, Basel, Switzerland
[6]Max Planck Institute for Terrestrial Microbiology, Karl-von-Frisch Strasse 10, 35043 Marburg, Germany
[7]Department of Biological Chemistry and Molecular Pharmacology, Harvard Medical School, 3 Blackfan Circle, Boston, MA 02115, USA
[8]Department of Pathology Department of Computational Medicine & Bioinformatics University of Michigan, Ann Arbor, MI, USA
[9]Walter and Elisa Hall Institute, 1G Royal Parade, Parkville, Victoria 3052, Australia
[10]These authors contributed equally
[11]Lead Contact
*Correspondence: pmeier@icr.ac.uk
https://doi.org/10.1016/j.celrep.2018.03.054

SUMMARY

Tumor necrosis factor (TNF) is an inflammatory cytokine that can signal cell survival or cell death. The mechanisms that switch between these distinct outcomes remain poorly defined. Here, we show that the E3 ubiquitin ligase Mind Bomb-2 (MIB2) regulates TNF-induced cell death by inactivating RIPK1 via inhibitory ubiquitylation. Although depletion of MIB2 has little effect on NF-κB activation, it sensitizes cells to RIPK1- and caspase-8-dependent cell death. We find that MIB2 represses the cytotoxic potential of RIPK1 by ubiquitylating lysine residues in the C-terminal portion of RIPK1. Our data suggest that ubiquitin conjugation of RIPK1 interferes with RIPK1 oligomerization and RIPK1-FADD association. Disruption of MIB2-mediated ubiquitylation, either by mutation of MIB2's E3 activity or RIPK1's ubiquitin-acceptor lysines, sensitizes cells to RIPK1-mediated cell death. Together, our findings demonstrate that Mind Bomb E3 ubiquitin ligases can function as additional checkpoint of cytokine-induced cell death, selectively protecting cells from the cytotoxic effects of TNF.

INTRODUCTION

TNF functions as a master regulator of the cytokine network that coordinates defense of homeostasis by controlling inflammation, cell proliferation, differentiation, survival, and death (Balkwill, 2009). Ubiquitylation has emerged as a crucial mediator of signal transduction in inflammation, stress responses, and defense of homeostasis (Dikic et al., 2009). The formation of atypical ubiquitin (Ub) chains produce robust signaling "hubs" that are recognized by specialized Ub-binding proteins (Husnjak and Dikic, 2012), which subsequently coordinate tissue repair and adaptation to tissue stress. The signaling pathways emanating from TNF receptor-1 (TNF-R1) represent some of the best models to study the role of Ub in the regulation of homeostasis. In mammals, binding of TNF to TNF-R1 triggers either pro-survival/inflammatory or pro-death signaling pathways (Walczak, 2013). TNF regulates tissue homeostasis by orchestrating three distinct signals: (1) the activation of NF-κB-dependent and MAPK/JNK-dependent transcriptional programs, (2) induction of caspase-8-dependent apoptosis, and (3) stimulation of receptor interacting protein kinase (RIPK)-mediated necrosis (necroptosis) (Declercq et al., 2009).

Binding of TNF to TNF-R1 results in the sequential formation of two signaling complexes (Walczak, 2011). The rapidly forming complex-I is assembled at the receptor's cytoplasmic tail and consists of the adaptor TRADD, RIPK1, TRAF2, cIAP1, and cIAP2. Within this complex, RIPK1 and other proteins are rapidly conjugated with Ub chains of various types. Using Jurkat, L929 cells, and mouse embryonic fibroblasts (MEFs), it was established that ubiquitylation of RIPK1 at K377 is indispensable for TNF-induced activation of NF-κB (Ea et al., 2006; Grunert et al., 2012; Vanlangenakker et al., 2011; Zhang et al., 2011). Since then, many additional studies have refined this viewpoint, demonstrating that the requirement of RIPK1 for NF-κB activation is cell type dependent (Blackwell et al., 2013; Suda et al., 2016; Wong et al., 2010). Together with LUBAC (linear Ub chain assembly complex; Tokunaga et al., 2009), complex-I signals

This is an open access article under the CC BY license (http://creativecommons.org/licenses/by/4.0/).

inflammation and cell survival through TAK1 and IκB kinase (IKK)-dependent activation of NF-κB, which drives production of cytokines as well as pro-survival genes such as *cFlip*.

Through a process still ill defined, complex-I dissociates from the receptor, and RIPK1 together with TRADD associates with the adaptor protein FADD and pro-caspase-8 (casp-8) to form complex-II (Micheau and Tschopp, 2003). The formation and activity of this signaling platform, which has the potential to initiate apoptosis or necroptosis, is tightly regulated by anti-apoptotic proteins such as cFLIP (Micheau et al., 2001; Panayotova-Dimitrova et al., 2013). In addition, NEMO and TAK1 also act as regulators of TNF-induced cell death, partly independent of their role in NF-κB activation (Bettermann et al., 2010; O'Donnell et al., 2007). The molecular mechanism underpinning the suppression of TNF-mediated complex-II formation is still poorly characterized. The current dogma dictates that ubiquitylation of RIPK1 mediates activation of NF-κB, cell survival and tissue repair, and its deubiquitylation, by de-ubiquitylating enzymes (DUBs) such as CYLD, enhances complex-II formation and caspase-8-mediated apoptosis or necroptosis (Walczak, 2011). It is believed that the Ub chains conjugated to RIPK1 by cIAP1/2 and LUBAC in complex-I constitute the decisive factor preventing RIPK1 from forming complex-II and limiting its killing potential (Bertrand et al., 2008; Gerlach et al., 2011; Haas et al., 2009). However, this was recently challenged by the observations that formation of complex-II also occurs under conditions where cIAPs and LUBAC are fully functional and following inhibition of TAK1 as well as loss of NEMO (Dondelinger et al., 2013; Kondylis et al., 2015; O'Donnell et al., 2007; O'Donnell and Ting, 2011; Panayotova-Dimitrova et al., 2013). Under such conditions, complex-II assembles despite RIPK1 ubiquitylation in complex-I. We and others have also recently identified MK2 as an additional checkpoint that regulates RIPK1 entry into complex-II. MK2 phosphorylates RIPK1 at Ser320 and Ser335 upon TNF stimulation to limit cell death downstream of TNF-R1 (Dondelinger et al., 2017; Jaco et al., 2017; Menon et al., 2017).

Because an inflammatory microenvironment can dictate the aggressiveness of certain tumors, and influence the response to therapy, a better understanding of the complex relationship between cell death and inflammation is critical. This is an important issue because its resolution, and putative therapeutic intervention, would allow the diversion of cancer-related inflammation into activation of cell death. Here, we report that Mind Bomb E3 Ub ligases contribute to the regulation of TNF-induced cell death by inactivating RIPK1 via inhibitory ubiquitylation.

RESULTS

Identification of MIB2 as a RIPK1-Binding Protein

To elucidate what determines whether an inflammatory signal instructs either an innate immune response or triggers cell death, we undertook a proteomic-based approach using components of the TNF-R signaling complex (TNF-RSC) as affinity reagents. Proteins of the TNF-RSC were fused to 2x-HA tags and expressed in Flp-In T-REx-HEK293 cells that allow single-copy insertion of these components under the control of doxycycline (Dox). HA-tagged proteins were purified and the presence of co-purified proteins was determined via mass spectrometry. In addition to known constituents of the native TNF-RSC, we identified the RING-type Ub-E3 ligase Mind Bomb-2 (MIB2) as a RIPK1-interacting protein (Figure 1A). Notably, MIB2 consistently co-purified with RIPK1 in six independent mass spectrometric experiments. We next verified the interaction of MIB2 with RIPK1 via co-immuno-precipitation and demonstrated that MIB2 readily interacted with RIPK1 (Figure 1B). MIB1, which shares 42% sequence homology with MIB2, also associated with RIPK1 under these conditions.

MIB2 Is a Constituent of the Native TNF-RSC

Consistent with the notion that MIB2 is part of complex-I, and in agreement with a recent mass spectrometry study (Wagner et al., 2016), we found that endogenous MIB2 was readily recruited to the TNF-RSC in a ligand- and time-dependent manner in a range of cell types, including MDA-MB-231, HT1080, and 786-0 (Figures 1C–1E). MIB2 recruitment was mainly RIPK1 dependent (Figure 1E) and occurred in the same dynamic manner as described for other components of complex-I (Gerlach et al., 2011; Haas et al., 2009; Micheau and Tschopp, 2003), peaking at 15 min. Reciprocal immuno-precipitation of endogenous MIB2, using MIB2-specific antibodies, likewise co-purified ubiquitylated RIPK1 and other components of complex-I such as TRADD, TNF-R1, and SHARPIN in a TNF- and time-dependent manner in multiple cell types (Figures 1F and 1G). This demonstrates that MIB2 is recruited to the initial complex-I that forms directly upon TNF stimulation. Although MIB2 is recruited to complex-I, our data indicated that in the cell lines tested, MIB2 had no role in TNF-induced activation of NF-κB, induction of NF-κB target genes such as A20, and the production of cytokines (Figures S1A–S1G).

MIB2 Protects Cells from TNF-Induced and RIPK1-Dependent Cell Death

Given that MIB2 did not modulate TNF-induced activation of NF-κB in the cell lines tested, we explored the role of this E3 ligase in regulating TNF-induced and RIPK1-dependent cell death. We tested a range of different cell lines that exhibit diverse sensitivities to TNF-induced cell death (Figures S2A–S2C) (Tenev et al., 2011; Vince et al., 2007). Specifically, we tested two paradigms of TNF-induced and RIPK1-dependent cell death, one that relies on the inhibition of TAK1 and one that occurs upon inactivation of IAPs with SMAC mimetic (SM) compounds. Although many cells are sensitive to TNF in the presence of the TAK1 kinase inhibitor 5Z-7-oxozeaenol (hereafter referred to as TAK1i), we focused our attention on a cell line that is largely resistant to this treatment combination, namely, the renal cell adenocarcinoma 786-0. Intriguingly, depletion of *MIB2*, but not *MIB1*, dramatically sensitized 786-0 cells to treatment with TNF in the presence of TAK1i, in both short-term cell death experiments and long-term clonogenic survival assays (Figures 2A–2C and S2D). Cell death under these conditions was entirely RIPK1 and caspase-8 dependent, as co-depletion of *MIB2* and *RIPK1* or *CASPASE-8* protected cells from the cytotoxic effects of TNF/TAK1i, and treatment with z-VAD-FMK completely suppressed cell death, corroborating the notion that these cells die by apoptosis (Figures 2B and S2D). In agreement with MIB2 limiting RIPK1- and caspase-8-dependent apoptosis,

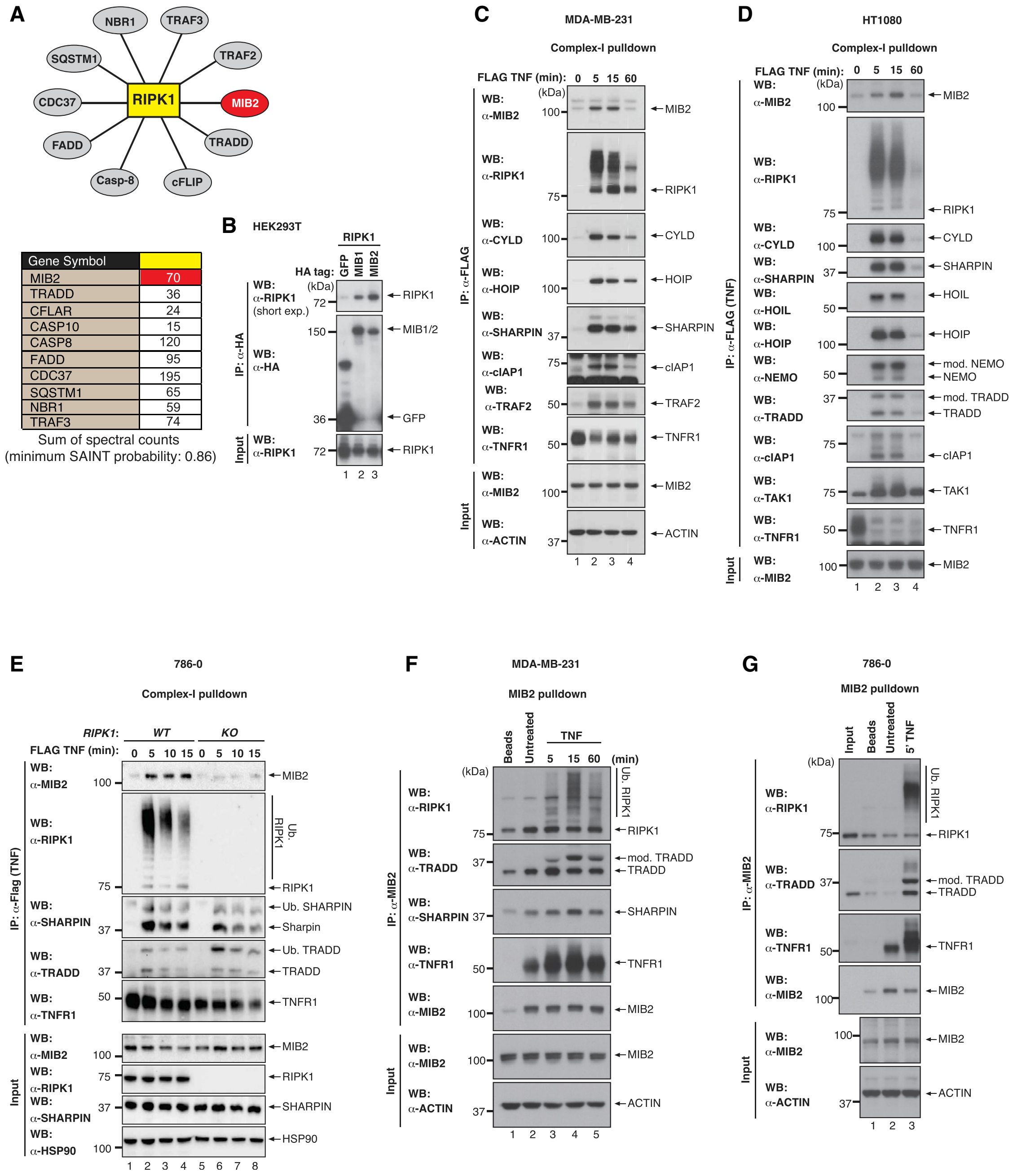

Figure 1. Identification of MIB2 as a Novel Component of TNF Receptor Complex-I

(A) Schematic representation of the RIPK1-bound target proteins that were identified by mass spectrometry. The table specifies the sum of the spectral counts across six independent immuno-precipitations for each target protein with a minimum SAINT score probability of 0.86.

(B) HA-GFP, HA-MIB1, or HA-MIB2 was co-expressed with untagged RIPK1 in 293T cells. HA-immuno-precipitation was performed and RIPK1 interaction was assessed via western blot.

(C–E) TNF-induced complex-I immuno-precipitation. MDA-MB-231 cells (C) or HT1080 cells (D) or WT 786-0 or *RIPK1* knockout (KO) 786-0 cells (E) were treated with FLAG-hTNF (0.8 μg/mL) for the indicated time points, followed by FLAG immuno-precipitation and western blot analysis.

(F and G) Western blot analysis of MDA-MB-231 cells (F) or 786-O cells (G) either left untreated or treated with FLAG-hTNF (0.8 μg/mL) for the indicated time points followed by MIB2 immuno-precipitation.

formation of complex-II was also enhanced upon *MIB2* knock-down (Figure 2D, top, compare lane 9 with lane 10). *MIB2* depletion also sensitized cells under conditions in which expression of NF-κB target genes were blocked by expressing a dominant-negative form of IκB (Super-Repressor; $I\kappa B^{SR}$) and to a lesser extent upon treatment with cycloheximide (CHX) (Figures S2E

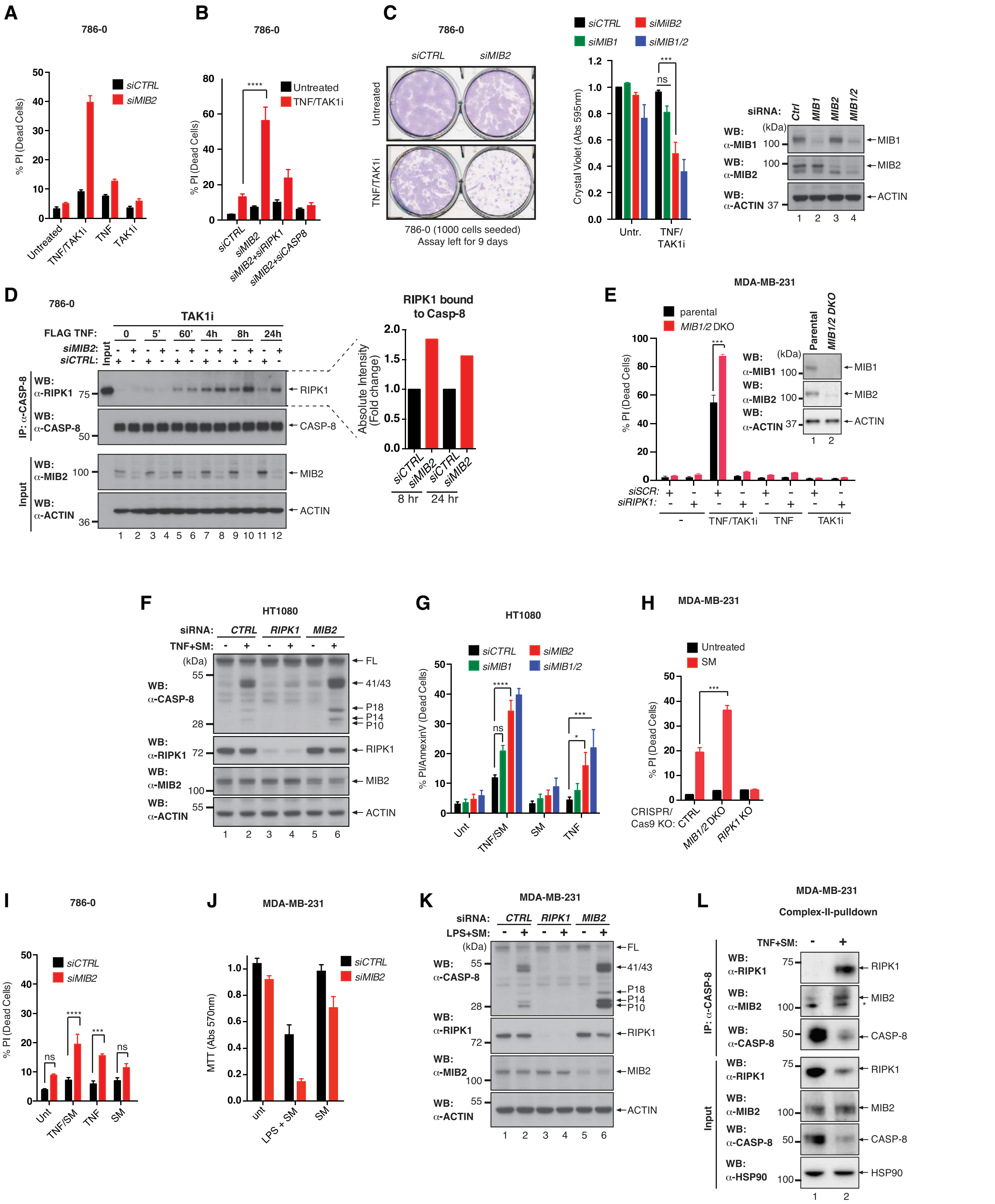

Figure 2. Depletion of MIB2 Sensitizes Cells to TNF-Induced and RIPK1-Dependent Cell Death

(A) FACS analysis of PI-positive 786-0 cells subjected to siRNA knockdown of *MIB2*. After siRNA knockdown, cells were treated with the indicated agents for 48 hr. Error bars represent SD.

(B) FACS analysis of PI-positive 786-0 cells subjected to siRNA knockdown of indicated genes. After siRNA knockdown, cells were treated with the indicated agents for 48 hr. Error bars represent SD.

(legend continued on next page)

and S2F). Moreover, CRISPR/Cas9-mediated deletion of *MIB1* and *MIB2* also sensitized the triple-negative breast cancer cell line MDA-MB-231 to TNF/TAK1i in a RIPK1-dependent manner (Figure 2E).

SM compounds block IAP proteins and trigger cytokine-dependent apoptosis in cancer cells. However, only a subset of cancer cells appears to be sensitive to SM treatment, and even sensitive cells can develop resistance. We therefore established the role of MIB proteins in modulating the therapeutic potential of SM compounds. Intriguingly, RNAi-mediated depletion or CRISPR/Cas9-mediated deletion of *MIB2* significantly enhanced activation of caspase-8 and the sensitivity of cells to TNF-induced cell death in the presence of SM in multiple cell types (Figures 2F–2L). Although RNAi-mediated knockdown of *MIB2* exacerbated the cytotoxic effects of TNF/SM, depletion of *MIB2* was somewhat sufficient to sensitize cells to TNF alone (Figures 2G and 2I). Interestingly, inhibiting the kinase activity of RIPK1 partially protected HT1080 and 786-0 cells from TNF-mediated cell death upon *MIB2* depletion (Figures S2G and S2H). Although the kinase activity of RIPK1 contributes to apoptosis in these cells, RIPK1's scaffolding function seemed to play the predominant role as depletion or genetic deletion of RIPK1 blocks cell death in these cells (Figures 2B, 2E, 2F, 2H, 2K, S2K, and S2L). Together, our data suggest that MIBs and IAPs both suppress TNF-dependent and RIPK1-mediated cell death.

Depletion of *MIB2* sensitized cells not only to exogenous TNF but also to autocrine and paracrine TNF, produced in response to treatment with lipopolysaccharide (LPS) that activates TLR4 and drives TLR4-mediated and NF-κB-dependent production of TNF (Figure 2K). Consistent with an increase in caspase activity (data not shown), we found that activation/cleavage of caspase-8 was enhanced upon knockdown of *MIB2* under these conditions (Figures 2J and 2K). Additionally, we found that MIB2 co-purified with complex-II (Figure 2L).

Some cells, such as the rhabdomyosarcoma cell line Kym1, were exclusively reliant on MIB2, as mere knockdown of *MIB2*, using two independent small interfering RNA (siRNA) oligos, induced cell death and loss of clonogenic growth potential (Figures S2I and S2J). Depletion of *MIB2* resulted in RIPK1-mediated formation of complex-II and activation of caspase-8 (Figures S2K and S2L). Genetic deletion of TNF-R1 almost completely abrogated the sensitivity of Kym1 cells to depletion of *MIB2* (Figure S2M). Together, these data indicate that MIB2 protects Kym1 cells from the cytotoxic potential of RIPK1.

To address the role of MIB2 in regulating necroptosis, we used murine SWISS-3T3 cells that can die by apoptosis or necroptosis. Of note, all other cell lines used in our study do not express detectable levels of RIPK3 and hence are refractory to necroptosis triggers. Although depletion of *Mib2* sensitized SWISS-3T3 cells to caspase-mediated and RIPK1 kinase-dependent apoptosis upon treatment with TNF/SM, knockdown of *Mib2* had no effect on necroptosis induced by TNF/SM/zVAD (Figures S2N and S2O). This suggests that at least in SWISS-3T3 cells, MIB2 selectively regulates apoptosis. It is important to note that in these cells, TNF/SM-mediated activation of caspases requires RIPK1 kinase activity. This is evident, as co-treatment with a selective RIPK1 kinase inhibitor blocks caspase activation upon TNF/SM treatment.

MIB2 Binds the Linker Region of Oligomeric RIPK1

Our data are consistent with the notion that MIB2 is a component of the TNF-RSC that contributes to the regulation of TNF-induced cell death. To further characterize the interaction of MIB2 with RIPK1, we generated a panel of deletion constructs for MIB2 (Figure 3A). We found that the N-terminal MZM region of MIB2 was necessary and sufficient for the interaction of MIB2 with RIPK1 (Figure 3B) and that deletion of the MZM region of MIB2 (Figure 3B), or mutation of the coordinating cysteine residue of the ZZ fold within the MZM region of MIB2 (Figure 3C), abrogated the interaction with RIPK1. We next generated deletion constructs for RIPK1 and tested their ability to bind to MIB2's MZM domain (Figure 3D). Of note, RIPK1 oligomerization is mediated by either the RHIM or DD domain. Deletion of both these oligomerization domains is required to interfere with RIPK1 homo-oligomerization (Figures S3A–S3F). We found that combined deletion of RIPK1's oligomerization surfaces (RHIM domain and DD, 1-510) abrogated the interaction of MIB2 with

(C) Clonogenic growth assay of 786-0 cells subjected to siRNA knockdown of indicated genes. 40 hr post-siRNA, 1,000 cells were re-plated, treated with TNF/TAK1i, and left in treated medium to form colonies. Error bars represent SEM. Western blot analysis of 786-0 cells subjected to *MIB1*, *MIB2*, or *MIB1/2* knockdown for 40 hr.

(D) Immuno-precipitation of complex-II following TNF stimulation. Cells were pre-treated with TAK1i and zVAD for 1 hr (zVAD and TAK1i also added to 0 hr) followed by treatment with FLAG-hTNF (0.8 μg/mL) for the indicated time points. Caspase-8 immuno-precipitation was performed followed by western blot analysis. Quantification of RIPK1 bound to caspase-8 is shown.

(E) FACS analysis of PI-positive *MIB1/2* DKO MDA-MB-231 cells subjected to siRNA knockdown of RIPK1 followed by treatment with TNF (10 ng/mL) or TAK1i (1 μM) alone or in combination for 16 hr. Error bars represent SD.

(F) Western blot analysis of activated caspase-8 (P41/43 cleavage product) following siRNA-mediated knockdown of *CTRL*, *RIPK1*, or *MIB2* in HT1080 cells and treatment with TNF/SM for 3 hr.

(G) FACS analysis of PI/AnnexinV-positive HT1080 cells subjected to siRNA knockdown of the indicated genes followed by treatment with TNF (10 ng/mL) or SM (100 nM) alone or in combination for 6 hr. Error bars represent SEM.

(H) FACS analysis of PI-positive *MIB1/2* DKO or *RIPK1* KO MDA-MB-231 cells treated with SM (100 nM) for 16 hr. Error bars represent SD.

(I) FACS analysis of PI-positive 786-0 cells treated with TNF (10 ng/mL) or SM (100 nM) or in combination for 48 hr. Error bars represent SD.

(J) MTT cell viability assay of MDA-MB-231 cells subjected to siRNA-mediated knockdown of *MIB2*. After siRNA-mediated knockdown, cells were treated with SM (100 nM) or LPS (10 μg/mL) alone or in combination for 3 hr. Error bars represent SD.

(K) Western blot analysis of activated caspase-8 (P41/43 cleavage product) following siRNA-mediated knockdown of *CTRL*, *RIPK1*, or *MIB2* in MDA-MB-231 cells and treatment with LPS/SM for 4.5 hr.

(L) TNF-induced complex-II immuno-precipitation. MDA-MB-231 cells were treated with the indicated agents for 4 hr. Caspase-8 immuno-precipitation was performed followed by western blot analysis. An asterisk indicates cross reactive bands.

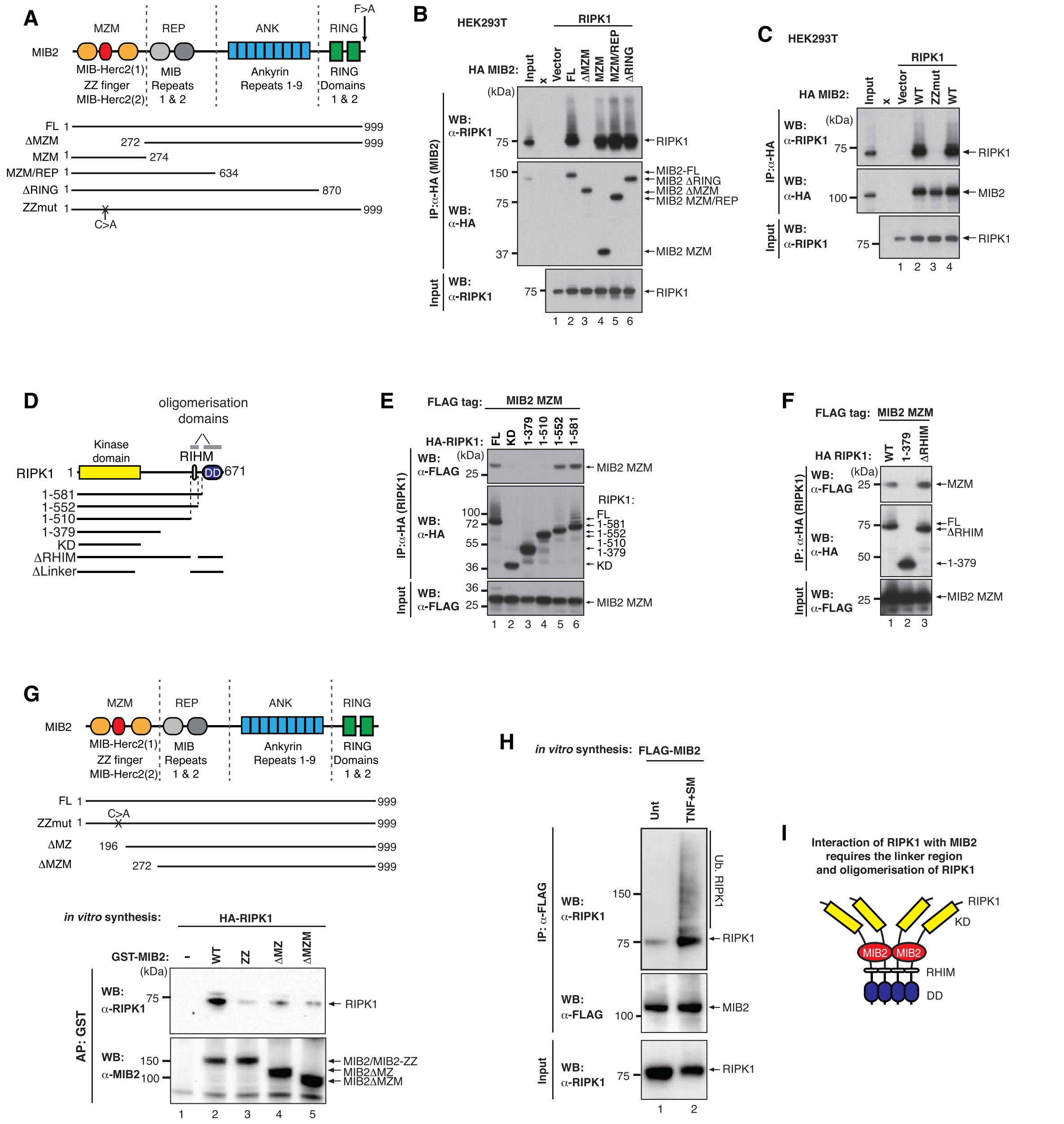

Figure 3. MZM Domain of MIB2 Is Required for Binding to Oligomeric RIPK1

(A) Domain organization of MIB2. The MZM, REP, ANK, and RING regions of the protein are indicated. The numbers depict the position of the amino acid of the indicated deletion constructs used.

(B) HA-MIB2 deletion constructs were co-expressed with untagged RIPK1 in 293T cells. HA-immuno-precipitation was performed and RIPK1 interaction was assessed via western blot.

(C) HA-vector control, HA-MIB2^{WT}, or HA-MIB2^{ZZ} (carrying a single point mutation in the ZZ-domain) was co-expressed with untagged RIPK1 in 293T cells. HA-immuno-precipitation was performed, and RIPK1 interaction was assessed via western blot.

(D) Schematic representation of RIPK1 constructs used in the binding studies.

(E and F) HA-RIPK1 deletion constructs (E) or HA-RIPK1ΔRHIM (F) were co-expressed with FLAG-MIB2 MZM domain in 293T cells. HA-immuno-precipitation was performed and interaction with MIB2 MZM was assessed via western blot.

(G) *In vitro* binding assay with recombinant MIB2 and *in vitro* translated RIPK1.

(H) Binding assay with MIB2 (*in vitro* translated) and RIPK1 (from cellular extract). MDA-MB-231 cells were left untreated or treated with TNF/SM/zVAD-fmk for 4 hr and lysates were incubated with *in vitro* translated FLAG-MIB2.

(I) Schematic representation of the interaction between MIB2 and oligomerized RIPK1.

RIPK1 (Figure 3E). Deletion of either the DD domain (1-581) on its own or removal of the RHIM in isolation (ΔRHIM) had no effect on the binding (Figures 3E and 3F). Although MIB2 did not bind to the kinase domain, it appeared to interact with the linker region of oligomerized RIPK1. Consistently, deletion of the linker region of RIPK1 abrogated MIB2 association (Figures 3F and S3D). Recombinant MIB2 and RIPK1 also bound to each other under *in vitro* conditions, indicating that this interaction is direct (Figures 3G and S3G). Under these conditions, *in vitro* translated RIPK1 oligomerized and was active (auto-phosphorylated at S166) (Figures S3H–S3J). Furthermore, we found that *in vitro* translated MIB2 was capable of purifying RIPK1 from cellular extract, particularly following treatment with TNF/SM (Figure 3H). Taken together, our data suggest that MIB2 interacts with the linker region of oligomerized RIPK1 (Figure 3I).

MIB2 Prevents RIPK1-Induced Death in a RING- and Binding-Dependent Manner

Although knockdown or genetic deletion of *MIB2* sensitized cells to caspase-8-mediated cell death, reconstitution with wild-type MIB2 suppressed the cytotoxic effects of TNF, as measured by caspase-8 maturation, generation of caspase activity (DEVDase assay), PARP cleavage, formation of complex-II, and viability assays (Figures 4A–4F). Reconstitution with $MIB2^{ZZ}$, which carries a mutation in the ZZ domain required for RIPK1 binding (Figures 4B, 4C, and 4E), failed to suppress TNF-induced caspase activation and cell death. Importantly, MIB2 with an amino acid substitution of the second-last Phe residue ($MIB2^{F>A}$) that abrogates its E3 ligase activity likewise failed to suppress TNF-induced cell death. Absence or mutational inactivation of MIB2 resulted in enhanced complex-II formation, caspase-8 activation, and cell death. Hence, physical association between MIB2 and RIPK1 is necessary but not sufficient to regulate RIPK1. This is evident because MIB2 RING finger mutants can still bind to RIPK1 (Figure 3B) but fail to regulate RIPK1-mediated activation of caspase-8 and cell death (Figures 4A–4F). These data suggest that after binding, MIB2 inhibits RIPK1 through a mechanism that is dependent on the E3 ligase activity of MIB2.

MIB2 Ubiquitylates RIPK1 upon TNF Stimulation Independently of cIAPs

Our data indicate that MIB2 is recruited to complex-I and suppresses the cytotoxic effect of RIPK1 in a RING finger-dependent manner. Consistent with the notion that RIPK1 is a physiological substrate of MIB2, we found that depletion or genetic deletion of *MIB2* resulted in a reduction in ubiquitylation of RIPK1 in complex-I in both MDA-MB-231 and 786-0 cells (Figures 5A and S4A). Although RNAi-mediated depletion or genetic deletion of *MIB2* resulted in reduced levels of ubiquitylated RIPK1 in complex-I, this had no apparent effect on the kinetics of the recruitment of other components of the TNF-RSC (Figure S4A) and did not affect TNF-induced activation of NF-κB (Figures 5B and S4B). Of note, MDA-MB-231, 786-0, and Kym-1 cells do not rely on RIPK1 for NF-κB activation and cytokine production (Figures 5B, 5C, S4B, and S4D), corroborating the notion that the requirement of RIPK1 for NF-κB activation is cell type dependent (Blackwell et al., 2013; Suda et al., 2016; Wong et al., 2010). Moreover, RIPK1 is not required for ERK, p38, and JNK activation downstream of TNF in 786-0 cells (Figure S4C). Although RIPK1 is required for NF-κB activation in Jurkat cells (Figure S4E; Ea et al., 2006), we were unable to investigate the role of MIB2 in RIPK1-mediated signaling downstream of TNF in Jurkat cells, because these cells do not survive long-term loss of MIB2 and are not sufficiently transfectable with siRNA oligos (data not shown). Following stimulation with TNF, MIB2 ubiquitylated RIPK1 in complex-I in a manner that was independent of IAPs. This is evident as the polyUb-smearing pattern of RIPK1 reproducibly increased in an IAP-independent yet MIB2-, TNF-, and time-dependent manner (Figures 5D–5F). Using the reconstitution system described in Figure 4A, we examined the requirement of the RING finger of MIB2 to promote RIPK1 ubiquitylation. Although wild-type MIB2 readily ubiquitylated RIPK1, the E3-Ub-deficient MIB2 mutant $MIB2^{F>A}$ failed to ubiquitylate RIPK1 (Figures 5E and 5F). MIB2 also ubiquitylated RIPK1 with the help of UbcH5a in an *in vitro* ubiquitylation assay (Figures 5G and S4G). Although MIB1 also ubiquitylated RIPK1, it was somewhat less efficient than MIB2 (Figure S4F).

MIB2 Conjugates Different Types of Ub Chains to RIPK1

To determine the Ub linkage types that are conjugated to RIPK1 by MIBs, we used UbiCRest (Ub chain architecture using Ub chain restriction; Hospenthal et al., 2015) analysis of RIPK1 in complex-I in the presence and absence of *MIB1/2*. Although the overall intensity of the ubiquitylation smearing pattern of RIPK1 was enhanced in the presence of endogenous MIB2 (Figure 6A), there was no apparent change in the Ub linkage repertoire. Accordingly, incubation with the respective DUBs resulted in a reduction of the Ub smearing pattern, indicating that K11-, K48-, and K63-linkage types were present. This demonstrates that K63-, K48-, and K11-linked chains were present in both *MIB1/2*-proficient as well as *MIB1/2*-deficient cells but are more abundant when MIBs are present. This suggests that endogenous MIBs can conjugate K11-, K48-, and K63-linked Ub chains to RIPK1. Using K-only Ub, we confirmed that MIB2 was indeed capable of conjugating K11-, K48-, and K63-linked polyUb chains to RIPK1 (Figure 6B).

MIB2 Ubiquitylates RIPK1 at Multiple Sites

To identify the lysine (K) residue(s) of RIPK1 that are ubiquitylated by MIB2, we used a semiquantitative mass spectrometry-based approach. In total, 14 K residues of RIPK1 were found to be modified by Ub (Figure 6C). These included previously reported K residues (green dots) (Ofengeim and Yuan, 2013) as well as several additional ubiquitylation sites on RIPK1 (yellow) (Figure 6C). The identified K residues of RIPK1 clustered into three regions: (1) kinase domain, (2) region surrounding K377, and (3) death domain (DD). Of the Ub-modified K residues, only four sites were specifically enriched by $MIB2^{WT}$ versus $MIB2^{F>A}$ (K316, K571, K604, and K634) (Figure 6D). K377 of human RIPK1, previously reported to be crucial for TNF-induced cell death (Ea et al., 2006; Li et al., 2006; O'Donnell et al., 2007), was not detected by mass spectrometry, because the tryptic digest generates peptide fragments that are too long for analysis. To address this issue, we conducted targeted mass spectrometry using double digestion with Trypsin and GluC. Although this approach successfully identified K377 of human RIPK1 as

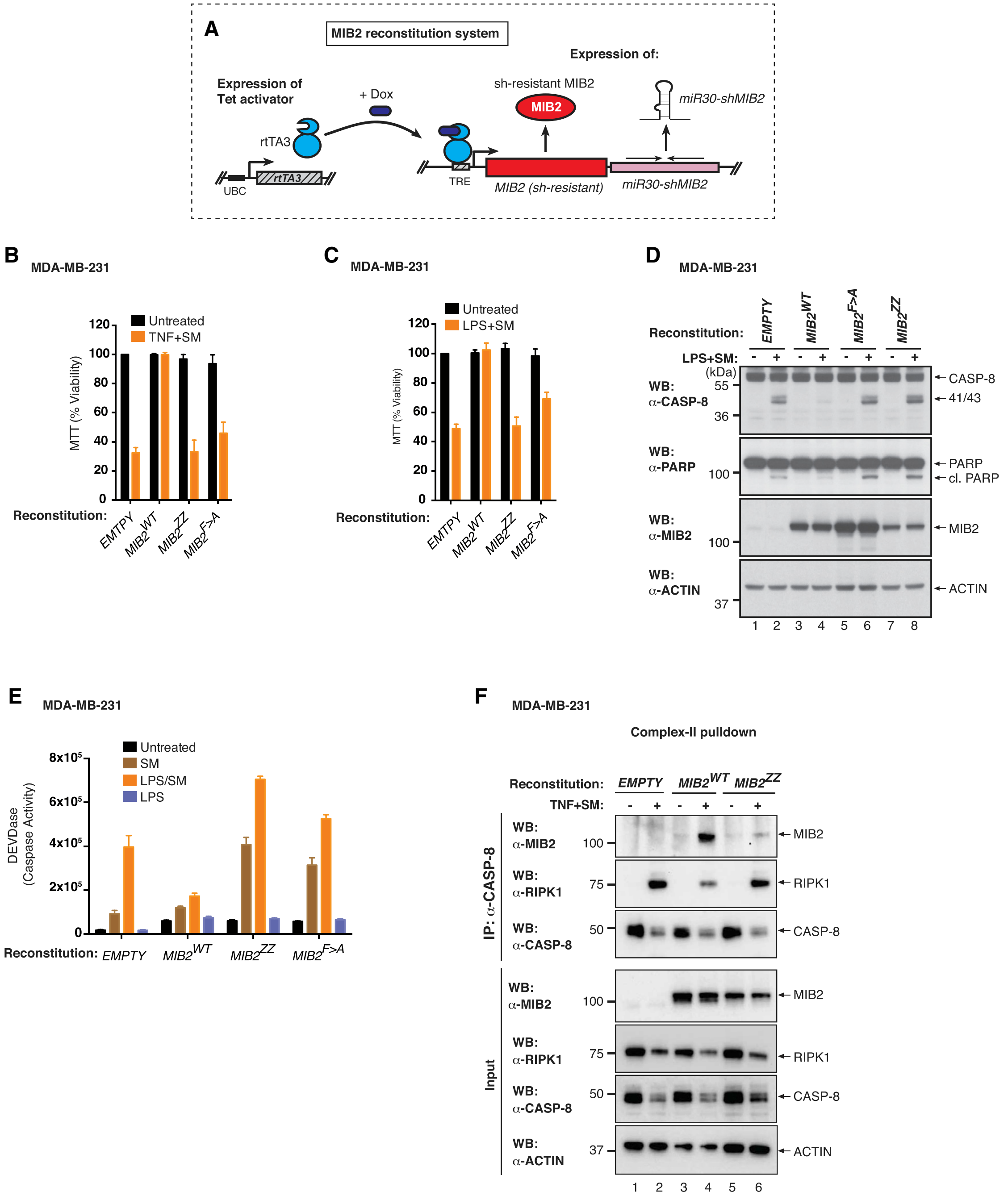

Figure 4. MIB2 Protects Cells from TNF-Induced and RIPK1-Dependent Cell Death, in a RING- and RIPK1-Binding-Dependent Manner

(A) Schematic diagram of the reconstitution system using MDA-MB-231 cells. Doxycycline treatment induces simultaneous knockdown of endogenous MIB2 and expression of shRNA resistant wild-type *MIB2*, $MIB2^{ZZ}$, $MIB2^{F>A}$, or *RFP*. TRE, tetracycline response element; UBC, ubiquitin promoter; rtTA3, reverse Tet transactivator.

(B and C) MTT cell viability assay of the indicated stable cells following induction of the target genes with doxycycline (500 ng/mL) for 64 hr and treatment with TNF (10 ng/mL) + SM (100 nM) for 3 hr (B) or with LPS (10 μg/mL) + SM (100 nM) for 5 hr (C). Error bars represent SD.

(D) Western blot analysis of activated caspase-8 (P41/43 cleavage product) using stable cells described in (A). Cells were induced for 64 hr with 500 ng/mL of doxycycline and treated ± LPS (10 μg/mL) + SM (100 nM) for 5 hr.

(E) DEVDase activity analysis using stable cells described in (A). Cells were induced for 64 hr with 500 ng/mL of doxycycline and treated with LPS (10 μg/mL) or SM (100 nM) alone or in combination for 3 hr. Error bars represent SD.

(F) TNF-induced complex-II immuno-precipitation. Stable cells described in (A) were induced with doxycycline for 64 hr and treated with the indicated agents for 4 hr. Caspase-8 immuno-precipitation was performed followed by western blot analysis.

OPEN ACCESS
CellPress

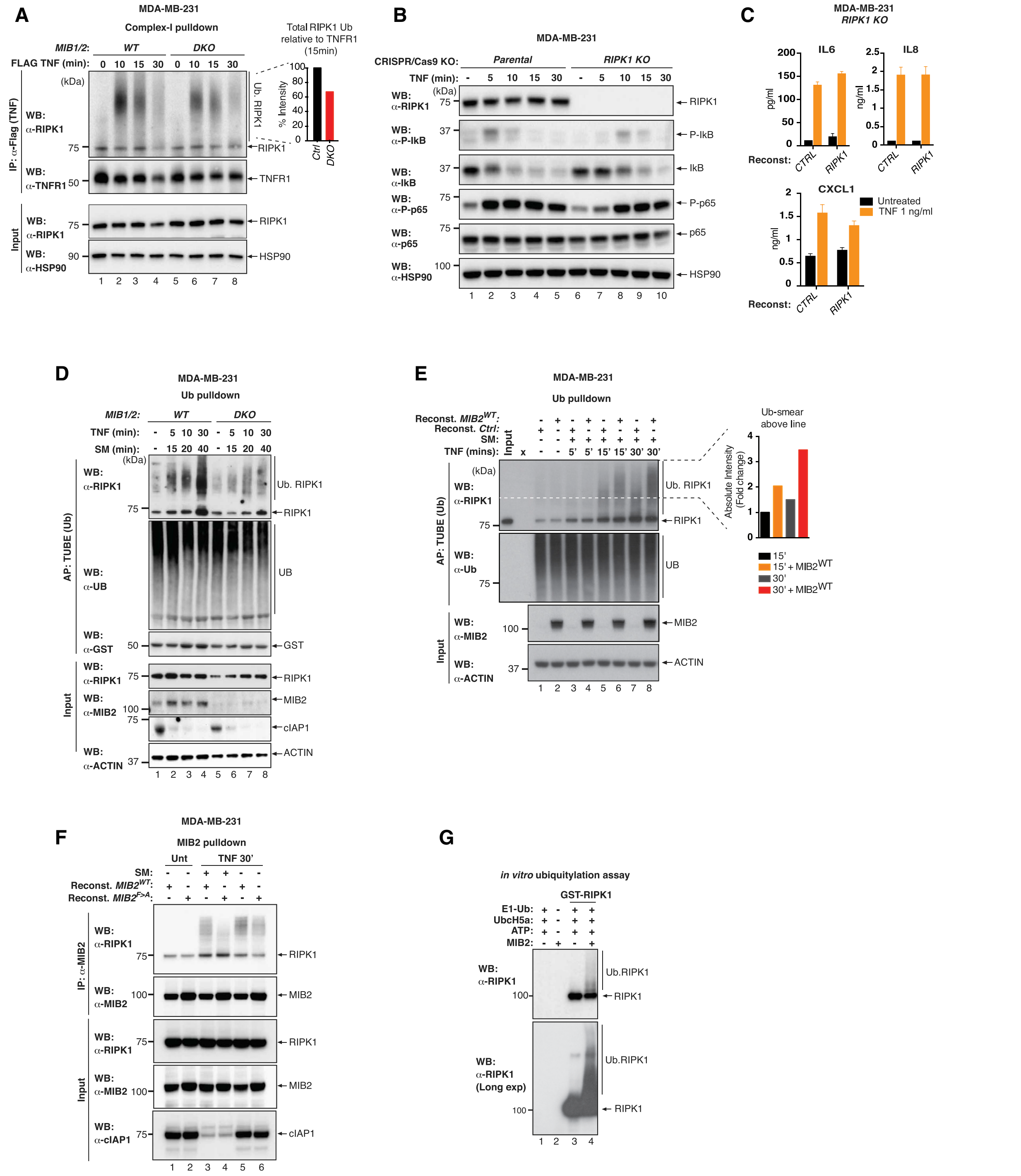

Figure 5. MIB2 Ubiquitylates RIPK1 in a TNF-Dependent Manner

(A) TNF-induced complex-I immuno-precipitation depicting the ubiquitylation of RIPK1. WT and *MIB1/2* DKO MDA-MB-231 cells were treated with FLAG-TNF (0.8 μg/mL) for the indicated time points, followed by FLAG immuno-precipitation and western blot analysis. Quantification for ubiquitylation is shown at 15 min time point.

(B) Comparison of TNF-induced NF-κB activation in parental and *RIPK1* KO MDA-MB-231 cells. Cells were either left untreated or treated with TNF (10 ng/mL) for indicated times and lysates were analyzed by western blotting

(C) The presence of cytokines in the culture media of parental and *RIPK1* KO MDA-MB-231 cells was determined by ELISA. Cells were stimulated with TNF for 6 hr. Error bars represent SD.

(legend continued on next page)

being ubiquitylated (Figure 6E), this approach did not allow accurate quantification of this ubiquitylation event. Whereas MIB2 readily ubiquitylated wild-type RIPK1, mutating K377 to R significantly reduced MIB2-mediated ubiquitylation of RIPK1 (Figure S5A). As expected, the ability of MIB2 to ubiquitylate K377 was critically dependent on MIB2's own RING finger activity, as the E3-deficient $MIB2^{F > A}$ mutant failed to ubiquitylate RIPK1 (Figure S5A). Although $RIPK1^{K377R}$ was less ubiquitylated in complex-I (Figure 6F), this mutant was significantly more auto-phosphorylated at S166 (Figure 6G). This indicates that ubiquitylation at K377 represses RIPK1's kinase activity. $RIPK1^{K377R}$ interacted with MIB2 as efficiently as $RIPK1^{WT}$, indicating that deregulation of $RIPK1^{K377R}$ was not due to impaired MIB2 binding (Figure S5B).

MIB2-Mediated Ubiquitylation of K377 and K634 Suppresses RIPK1's Cytotoxic Potential

Of the identified residues, K604 and K634 are positioned at the interface between two RIPK1 DD (type I and II interfaces) (Figures 7A–7D). Given their position, ubiquitylation of these residues is predicted to affect RIPK1 self-association. K634 also locates between RIPK1's DD and FADD's DD (type II interface), which is predicted to directly affect the interaction if ubiquitylated. Consistent with the structural prediction, we found that reconstituted $RIPK1^{K634R}$ cells were considerably more sensitive than $RIPK1^{WT}$ cells when challenged with TNF/TAK1i (Figures 7E, 7F, and S6). $RIPK1^{K634R}$ was as cytotoxic as $RIPK1^{K377R}$, indicating that ubiquitylation of either of these two sites represses the killing potential of RIPK1. Consistent with the notion that MIB2 regulates RIPK1 by targeting multiple K residues, depletion of MIB2 further reduced ubiquitylation of $RIPK1^{K634R}$ and $RIPK1^{K377R}$ (Figures S7A and S7B) and further sensitized $RIPK1^{K377R}$ cells to TNF killing (Figure 7G). Together, these data indicate that MIB2 antagonizes the lethal effects of TNF by attaching Ub chains to the linker and C-terminal portion of RIPK1, thereby skewing TNF's signaling potential toward pro-survival and pro-inflammation rather than cell death.

DISCUSSION

Ub-mediated inactivation of RIPK1 has long been postulated to contribute to the regulation of cytokine-induced cell death (Ea et al., 2006; Moquin et al., 2013; O'Donnell et al., 2007). cIAP- and LUBAC-mediated ubiquitylation of RIPK1 is a decisive factor in limiting the formation of complex-II (Bertrand et al., 2008; Gerlach et al., 2011; Haas et al., 2009). In the absence of either cIAPs or LUBAC or inhibition of the TAK1-IKKβ axis, TNF fails to activate canonical NF-κB effectively, and consequently, the levels of NF-κB target genes that limit complex-II activity, such as $cFLIP_L$, are insufficient to prevent caspase-8-mediated cell death. Primarily this occurs via the induction of NF-κB and the upregulation of cFLIP, but recent evidence suggests that TAK1 and IKK can regulate TNF killing independent of their role in NF-κB activation (Bettermann et al., 2010; Dondelinger et al., 2015; O'Donnell et al., 2007). In the absence of functional TAK1 and IKK, lethal levels of complex-II assemble despite RIPK1 ubiquitylation in complex-I (Dondelinger et al., 2013, 2015; Legarda-Addison et al., 2009). Under these conditions, TNF-mediated RIPK1-dependent apoptosis was shown to rely on the kinase activity of RIPK1 (Dondelinger et al., 2013; Wang et al., 2008). Thus, cells regulate TNF-induced cell death through multiple mechanisms.

Here, we have identified MIB2 as an E3 ligase that can regulate TNF killing. We find that it selectively regulates TNF-induced cell death, while it plays no role in NF-κB activation in the systems tested. MIB2 directly binds to RIPK1 and conjugates inhibitory Ub chains to the linker and the C-terminal portion of RIPK1. Although it remains to be determined how ubiquitylation of K377 suppresses RIPK1 kinase activity (auto-phosphorylation), ubiquitylation of K604 and K634 is predicted to affect RIPK1 self-association, as these residues are positioned at the interface between two RIPK1 DDs. Moreover, K634 is located between RIPK1's DD and FADD's DD, which is predicted to directly affect the interaction if ubiquitylated.

Different cells have different sensitivities to modulation of cIAPs and MIB2. Some cells are acutely sensitive to TNF-induced cell death in the absence of cIAPs or MIB2, while others are not. Consistently, while RNAi-mediated knockdown of MIB2 exacerbates the cytotoxic effects of TNF/SM, depletion of MIB2 is sufficient to sensitize certain cells to TNF alone (Figures 2G and 2I). The efficiency with which TNF/*siMIB2* induces cell death is comparable with that triggered by TNF/SM (Figures 2G and 2I). As cIAPs not only control TNF killing but also are key mediators of TNF-induced activation of NF-κB and the expression of pro-survival genes (Silke and Meier, 2013), their role clearly extends beyond simple regulation of RIPK1's cytotoxic potential. In contrast, MIB2 appears to exclusively inhibit RIPK1's killing potential. The notion that MIB2 contributes to the resistance to RIPK1-mediated cell death is also supported by the observation that high levels of MIB2 inhibit cell death triggered by TNF, SM, TNF/SM, or TNF/TAK1i. Because pharmacological inhibitors of IAPs are in clinical testing, our data suggest that targeting the MIB2 Ub ligase might improve the efficacy of SMs for the treatment of cancer. However, it should be noted that resistance to SM

(D) TUBE affinity purification of lysates from WT and *MIB1/2* DKO MDA-MB-231 cells pre-treated with SM (100 nM) for 10 min, followed by TNF (10 ng/mL) for the indicated time points.

(E) TUBE affinity purification of lysates from reconstituted MDA-MB-231 cells. Expression of the indicated proteins was induced with doxycycline (500 ng/mL) for 64 hr followed by pre-treatment with SM (100 nM) for 10 min, after which TNF (100 ng/mL) was added for the indicated times. Quantification of ubiquitylated RIPK1 above the dotted line at 15 and 30 min.

(F) Western blot analysis of reconstituted MDA-MB-231 cells. Prior treatment, expression of the indicated proteins was induced with doxycycline (500 ng/mL) for 60 hr. Cells were either left untreated or treated with TNF (100 ng/mL) in the presence or absence of SM (100 nM) for 30 min followed by MIB2 immunoprecipitation.

(G) *In vitro* ubiquitylation assay using the indicated purified proteins. The presence of ubiquitylated RIPK1 was evaluated by immunoblotting the reaction with an anti-RIPK1 antibody.

OPEN ACCESS CellPress

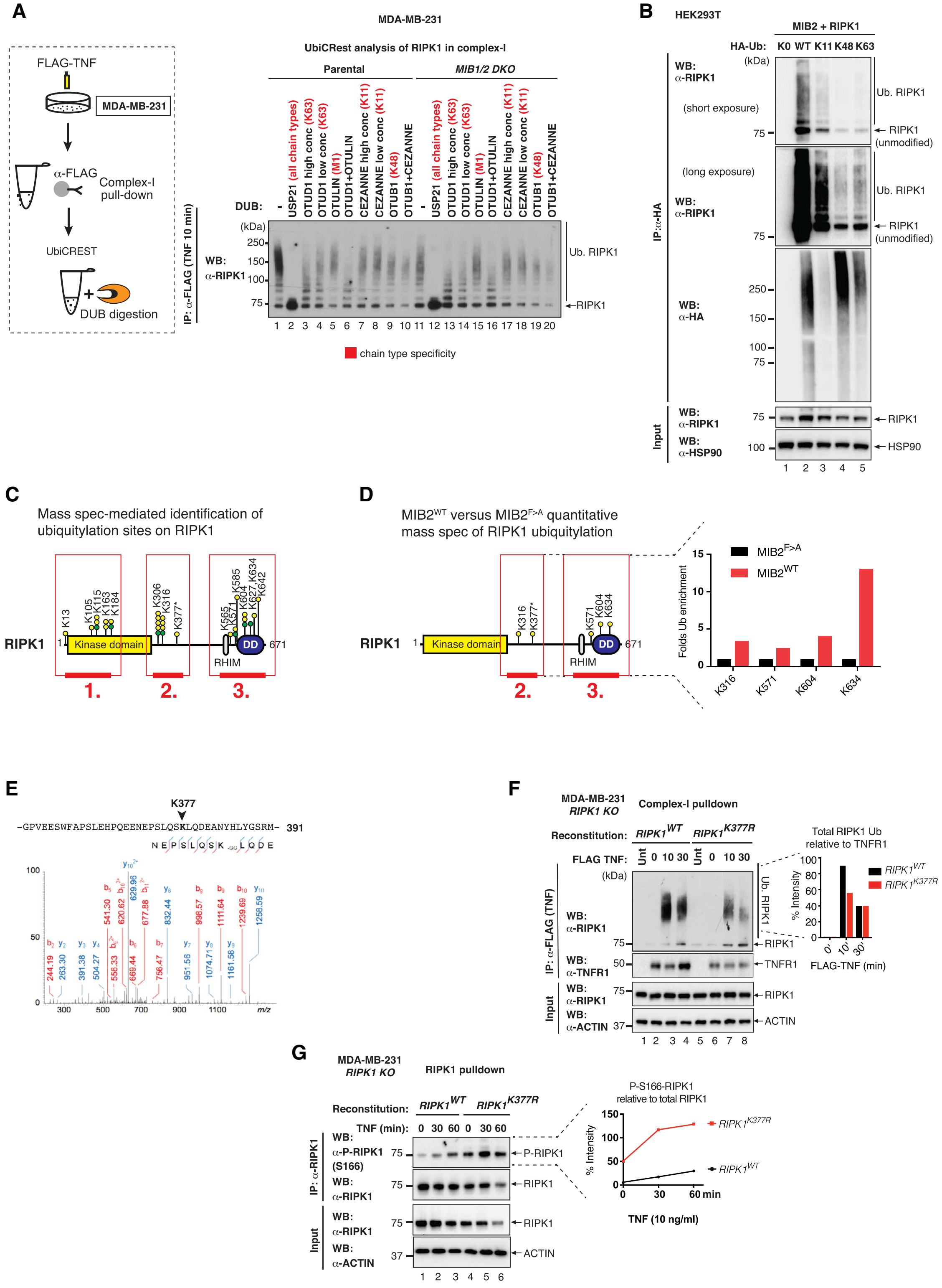

Figure 6. MIB2 Ubiquitylates the Linker and the C-Terminal Portion of RIPK1 with Different Types of Linkages

(A) UbiCRest analysis of ubiquitylated RIPK1 associated to complex-I. WT and *MIB1/2* DKO MDA-MB-231 cells were treated with FLAG-TNF (0.8 μg/mL) for 10 min, and complex-I was immuno-precipitated using FLAG beads. Beads were aliquoted and treated with the indicated DUBs in parallel reactions for 30 min at 37°C. The ubiquitylation status of RIPK1 was analyzed by western blot.

(legend continued on next page)

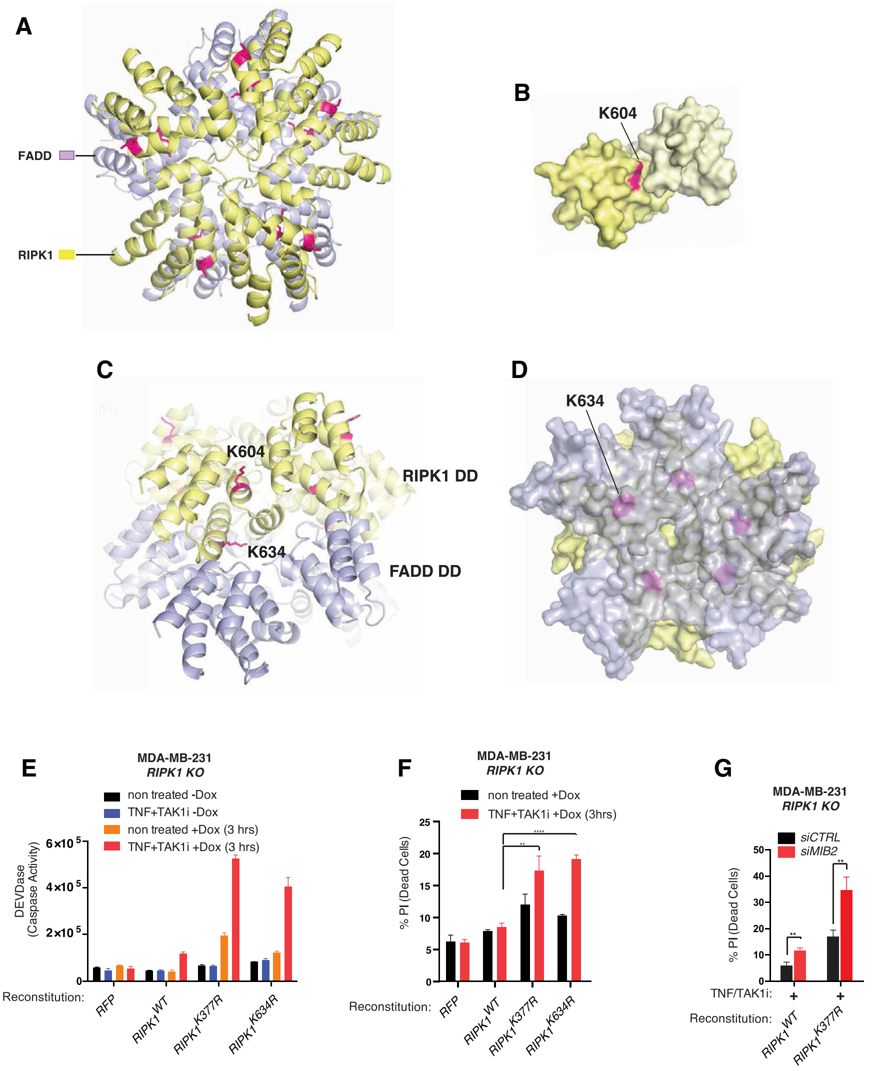

Figure 7. MIB2 Ubiquitylates RIPK1 at Lysines K377 and K634

(A–D) A homology model of human RIPK1 death domain (DD) (583–669) was generated by the SWISS-MODEL server. The PIDD DD (PDB: 2OF5) was used as homology template. RIPK1 DD model was aligned to Fas DD in Fas/FADD complex structure (PDB: 3OQ9) (A). Residues K604 (B) and K634 (C) are found at the interface between two RIPK1 DD (type I and II interfaces), which should affect RIPK1 self-association if ubiquitinated. K634 also locates between RIPK1 DD and FADD DD (type II interface) (D), which is predicted to directly affect the interaction if ubiquitylated.

(E) DEVDase activity assay of *RIPK1* KO MDA-MDA-MB-231 cells reconstituted with *RFP*, *RIPK1^{WT}*, *RIPK1^{K377R}*, or *RIPK1^{K634R}* and treated with TNF/TAK1i for 3 hr. Expression of *RFP*, *RIPK1^{WT}*, *RIPK1^{K377R}*, or *RIPK^{K634R}* was induced with doxycycline for 3 hr. Error bars represent SD.

(F) FACS analysis of PI-positive *RIPk1* KO MDA-MB-231 cells reconstituted with either RFP, *RIPK1^{WT}*, *RIPK1^{K377R}*, or *RIPK1^{K634R}* and treated with TNF/TAK1i for 3 hr. Error bars represent SD.

(G) FACS analysis of PI-positive MDA-MB-231 *RIPK1 KO* cells reconstituted with *RIPK1^{WT}* or *RIPK1^{K377R}* following MIB2 knockdown. Cells were stimulated with TNF/TAK1i for 3 hr. Error bars represent SD.

treatment can occur in multiple ways. Although levels of MIB2 clearly matter with regard to SM sensitivity, cells can also become resistant to SM by loss of autocrine-TNF, TNF-R1, RIPK1, FADD, caspase-8, and other mechanisms.

The importance of the E3 ligase activity of MIB2 in regulating RIPK1 and TNF-induced cell death, is highlighted by MIB2 variants bearing RING mutations. The ability of MIB2 to protect from the cytotoxic effects of TNF is strictly dependent on MIB2 neutralizing RIPK1 in a binding- and Ub-dependent manner. MIB2 mutations that selectively abrogate binding or ubiquitylation of RIPK1 cause loss of MIB2 function. The ability of MIB2 to bind to RIPK1 depends on activation of TNF-R1. This may be because TNF-mediated clustering of TNF-R1 results in oligomerization of RIPK1, which in turn is essential for MIB2 to bind and ubiquitylate RIPK1. In the absence of TNF-R1 activation, MIB2 does not associate with RIPK1, potentially because HSP90 sequesters RIPK1 in its monomeric, kinase inactive state (Lewis et al., 2000). Thus, under resting conditions, RIPK1 seems to reside in an inactive configuration that precludes MIB2 binding. Only when it is recruited to TNF-R1 can it bind tightly to MIB2. In this respect, MIB2 may sense the activity status of RIPK1.

(B) MIB2 can target RIPK1 for ubiquitylation with K11-, K48-, and K63-linked chains. The indicated constructs were co-expressed in HEK293T cells, and lysates were immunoblotted with an anti-RIPK1 antibody.

(C) Schematic representation of all ubiquitylation sites of RIPK1 identified by mass spectrometry-based experiments. Each circle indicates an independent experiment. Green marks previously identified residues.

(D) Schematic representation of the ubiquitylation sites of RIPK1 that are mediated by MIB2. Fold Ub enrichment of RIPK1 sites in MIB2^{WT} compared with MIB2$^{F > A}$ is shown.

(E) diGly MS/MS spectra of K377 of human RIPK1 (precursor ion 688.0078^{3+}, error -0.58 ppm, ion score 21.8). For clarity, only prominent fragment ions are marked.

(F) Ubiquitylation of RIPK1 in complex-I. *RIPK1* KO MDA-MB-231 cells reconstituted with either *RIPK1^{WT}* or *RIPK1^{K377R}* were treated with FLAG-hTNF for the indicated time points, followed by FLAG immune-precipitation and western blot analysis. Quantification for RIPK1 ubiquitylation is shown.

(G) Immuno-precipitation of RIPK1 from *RIPK1* KO MDA-MB-231 cells reconstituted with either *RIPK1^{WT}* or *RIPK1^{K377R}*. Cells were treated with FLAG-hTNF for the indicated time points, followed by RIPK1 immuno-precipitation and western blot analysis. Quantification for RIPK1 phosphorylation is shown.

This is similar to cIAPs, which also only regulate RIPK1 upon cytokine stimulation (Varfolomeev et al., 2007; Vince et al., 2007).

The question remains why there is an apparent overlap in the functions of MIB2 and cIAPs. One possible scenario might be that MIB2 and cIAPs target different K residues of RIPK1. It is interesting that MIB2 preferentially ubiquitylates the linker and the C-terminal portion of RIPK1, while other E3 ligases prefer to ubiquitylate the kinase domain (Figure 6C), albeit TRAF2/cIAPs also reportedly ubiquitylate K377 (O'Donnell et al., 2007), which seems to be a prominent Ub acceptor K. MIB2-mediated ubiquitylation of RIPK1 appears to interfere with RIPK1 oligomerization (DD-DD interactions), while cIAP-dependent RIPK1 ubiquitylation may suppress its kinase activity, which is required for complex-II formation in some cell types (Dondelinger et al., 2013; Wang et al., 2008).

Together, our data demonstrate that MIB2 contributes to inhibiting the pro-apoptotic activity of RIPK1. Although MIB2 is clearly not the only E3 ligase that can target RIPK1 for ubiquitylation, it is unique in its ability to selectively limit TNF-induced killing without affecting activation of TAK1, IKK, p38, JNK, and NF-κB in our system. Other E3 ligases in complex-I, such as cIAPs and LUBAC, influence TAK1 and IKK activation. Through ubiquitylation of RIPK1, MIB2 seems to consolidate the ubiquitylation status of RIPK1 in complex-I, thereby suppressing its transition to complex-II. The absence of MIB2 therefore specifically converts the predominant survival signal that originates from TNF-R1 into a death signal, a situation that might be relevant upon viral infection (pattern recognition receptor activation) or engagement of multiple cytokine receptors (Geserick et al., 2009; Tenev et al., 2011).

EXPERIMENTAL PROCEDURES

Further details and an outline of resources used in this work can be found in Supplemental Experimental Procedures.

Cell Culture

HT1080$^{IkB\text{-}SR}$, Kym1, HT1080, MDA-MB-231, Jurkat, SWISS-3T3, HEK293T, and Flp-In-REx-HEK293 cells were cultured in DMEM; 786-0 cells were cultured in RPMI-1640 medium. Culture media were supplemented with 10% fetal bovine serum (GIBCO), penicillin, and streptomycin (GIBCO), and all cells were cultured at 37°C with 10% CO_2 in a humidified incubator.

Generation of CRISPR Cells

Guide RNAs (gRNAs) were designed according to the Church lab (Mali et al., 2013) or Zhang lab (Ran et al., 2013). Prior to CRISPR/Cas9 editing, cells were sorted using fluorescence-activated cell sorting (FACS), and single clones were isolated and characterized. Single-cell clones were then transfected with Cas9 and gRNA targeting the gene of interest using Invitrogen reagents. Three days after transfection, cells were FACS-sorted, and single clones were isolated and screened for the deletion of the gene of interest. *MIB2* knockout clones or were further used to generate *MIB1/2* knockout cells. Positive clones were characterized. Guide sequences are available upon request.

Immuno-Precipitation Assays

Immuno-precipitation assays were performed as previously described (Jaco et al., 2017). For all immuno-precipitation assays cells were treated as indicated and lysed on ice in DISC lysis buffer supplemented with protease inhibitors. Cell lysates were rotated at 4°C for 20 min and then clarified at 4°C at 14,000 rpm for 10 min. Anti-FLAG M2 beads (20 μL) (Sigma), 20 μL of anti-HA beads (Sigma), or 20 μL of protein A/G agarose (Pierce) + MIB2 antibody (Bethyl Laboratories) or RIPK1 BD antibody; 1 μg antibody/mg protein lysate were rotated with cleared protein lysates overnight at 4°C. Washes (4×) in wash buffer (50 mM Tris [pH 7.5], 150 mM NaCl, 0.1% Triton X-100, and 5% glycerol) were performed, and samples eluted by boiling in 50 μL 1× SDS loading dye.

Statistics

Statistical analysis was performed using GraphPad Prism version 6.0. Unless otherwise specified, data are presented as mean ± SEM. Comparisons were performed using a Student's t test whose values are represented in the figures as $*p < 0.05$, $**p < 0.01$, and $***p < 0.001$.

SUPPLEMENTAL INFORMATION

Supplemental Information includes Supplemental Experimental Procedures and seven figures and can be found with this article online at https://doi.org/10.1016/j.celrep.2018.03.054.

ACKNOWLEDGMENTS

We would like to thank Henning Walczak, Freddy Radtke, Shaomeng Wang, Patricia J. Gallagher, Vanessa Redecke, Katrin Rittinger, and Ivan Dikic for reagents and advice. We also thank members of the Komander and Meier labs for helpful discussions. We would like to apologize to the many authors whose work we could not cite because of space restrictions. Work in the Meier lab is funded by Breast Cancer Now (CTR-QR14-007), the Medical Research Council (MRC) (MR/M019217/1), Komen Promise (PG12220321), Worldwide Cancer Research (15-0042), Biotechnology and Biological Sciences Research (BBSRC) (BB/L021684/1), and postgraduate studentships from Cancer Research UK (CRUK) (CRM089X). The Martin laboratory is supported by PI (08/IN.1/B2031) and SRC (07/SRC/B1144) grants from Science Foundation Ireland. C.J.K. was supported by an Irish Research Council for Science Engineering and Technology (IRCSET) postgraduate studentship (RS/2010/2542). The Nesvizhskii lab is supported by the NIH (GM094231 and CA126239). The Wu lab is funded by the NIH (AI050872 to H.W.). Work in the Komander lab is funded by the MRC (U105192732), the European Research Council (724804), and the Lister Institute of Preventive Medicine. We acknowledge National Health Service (NHS) funding to the National Institute for Health Research (NIHR) Biomedical Research Centre.

AUTHOR CONTRIBUTIONS

R.F., K.J., and T.T. contributed equally to this study and are listed alphabetically. P.M., R.F., K.J., and T.T. designed the research and wrote the manuscript. R.F., K.J., and T.T. performed experiments. G.L. performed the PLA assay. I.J. performed some death assays. O.M. and C.M.D. conducted preliminary experiments using *Drosophila* melanogaster. S.W.J. performed the Y2H assay. A.A, T.G., A.N., A.S., M.W., and A.J.T. conducted the mass spectrometry analysis. C.J.K., D.C., and S.M. conducted the cytokine analysis. A.A., P.R.E., and D.K. helped with the UbiCRest analysis. Q.Q. and H.W. did the structural homology model.

DECLARATION OF INTERESTS

The authors declare no competing interests.

Received: October 17, 2017
Revised: January 16, 2018
Accepted: March 13, 2018
Published: April 10, 2018

REFERENCES

Balkwill, F. (2009). Tumour necrosis factor and cancer. Nat. Rev. Cancer *9*, 361–371.

Bertrand, M.J., Milutinovic, S., Dickson, K.M., Ho, W.C., Boudreault, A., Durkin, J., Gillard, J.W., Jaquith, J.B., Morris, S.J., and Barker, P.A. (2008).

cIAP1 and cIAP2 facilitate cancer cell survival by functioning as E3 ligases that promote RIP1 ubiquitination. Mol. Cell *30*, 689–700.

Bettermann, K., Vucur, M., Haybaeck, J., Koppe, C., Janssen, J., Heymann, F., Weber, A., Weiskirchen, R., Liedtke, C., Gassler, N., et al. (2010). TAK1 suppresses a NEMO-dependent but NF-kappaB-independent pathway to liver cancer. Cancer Cell *17*, 481–496.

Blackwell, K., Zhang, L., Workman, L.M., Ting, A.T., Iwai, K., and Habelhah, H. (2013). Two coordinated mechanisms underlie tumor necrosis factor alpha-induced immediate and delayed IκB kinase activation. Mol. Cell. Biol. *33*, 1901–1915.

Declercq, W., Vanden Berghe, T., and Vandenabeele, P. (2009). RIP kinases at the crossroads of cell death and survival. Cell *138*, 229–232.

Dikic, I., Wakatsuki, S., and Walters, K.J. (2009). Ubiquitin-binding domains—from structures to functions. Nat. Rev. Mol. Cell Biol. *10*, 659–671.

Dondelinger, Y., Aguileta, M.A., Goossens, V., Dubuisson, C., Grootjans, S., Dejardin, E., Vandenabeele, P., and Bertrand, M.J. (2013). RIPK3 contributes to TNFR1-mediated RIPK1 kinase-dependent apoptosis in conditions of cIAP1/2 depletion or TAK1 kinase inhibition. Cell Death Differ. *20*, 1381–1392.

Dondelinger, Y., Jouan-Lanhouet, S., Divert, T., Theatre, E., Bertin, J., Gough, P.J., Giansanti, P., Heck, A.J., Dejardin, E., Vandenabeele, P., and Bertrand, M.J. (2015). NF-κB-independent role of IKKα/IKKβ in preventing RIPK1 kinase-dependent apoptotic and necroptotic cell death during TNF signaling. Mol. Cell *60*, 63–76.

Dondelinger, Y., Delanghe, T., Rojas-Rivera, D., Priem, D., Delvaeye, T., Bruggeman, I., Van Herreweghe, F., Vandenabeele, P., and Bertrand, M.J.M. (2017). MK2 phosphorylation of RIPK1 regulates TNF-mediated cell death. Nat. Cell Biol. *19*, 1237–1247.

Ea, C.K., Deng, L., Xia, Z.P., Pineda, G., and Chen, Z.J. (2006). Activation of IKK by TNFalpha requires site-specific ubiquitination of RIP1 and polyubiquitin binding by NEMO. Mol. Cell *22*, 245–257.

Gerlach, B., Cordier, S.M., Schmukle, A.C., Emmerich, C.H., Rieser, E., Haas, T.L., Webb, A.I., Rickard, J.A., Anderton, H., Wong, W.W., et al. (2011). Linear ubiquitination prevents inflammation and regulates immune signalling. Nature *471*, 591–596.

Geserick, P., Hupe, M., Moulin, M., Wong, W.W., Feoktistova, M., Kellert, B., Gollnick, H., Silke, J., and Leverkus, M. (2009). Cellular IAPs inhibit a cryptic CD95-induced cell death by limiting RIP1 kinase recruitment. J. Cell Biol. *187*, 1037–1054.

Grunert, M., Gottschalk, K., Kapahnke, J., Gündisch, S., Kieser, A., and Jeremias, I. (2012). The adaptor protein FADD and the initiator caspase-8 mediate activation of NF-κB by TRAIL. Cell Death Dis. *3*, e414.

Haas, T.L., Emmerich, C.H., Gerlach, B., Schmukle, A.C., Cordier, S.M., Rieser, E., Feltham, R., Vince, J., Warnken, U., Wenger, T., et al. (2009). Recruitment of the linear ubiquitin chain assembly complex stabilizes the TNF-R1 signaling complex and is required for TNF-mediated gene induction. Mol. Cell *36*, 831–844.

Hospenthal, M.K., Mevissen, T.E.T., and Komander, D. (2015). Deubiquitinase-based analysis of ubiquitin chain architecture using Ubiquitin Chain Restriction (UbiCRest). Nat. Protoc. *10*, 349–361.

Husnjak, K., and Dikic, I. (2012). Ubiquitin-binding proteins: decoders of ubiquitin-mediated cellular functions. Annu. Rev. Biochem. *81*, 291–322.

Jaco, I., Annibaldi, A., Lalaoui, N., Wilson, R., Tenev, T., Laurien, L., Kim, C., Jamal, K., Wicky John, S., Liccardi, G., et al. (2017). MK2 phosphorylates RIPK1 to prevent TNF-induced cell death. Mol. Cell *66*, 698–710.e5.

Kondylis, V., Polykratis, A., Ehlken, H., Ochoa-Callejero, L., Straub, B.K., Krishna-Subramanian, S., Van, T.M., Curth, H.M., Heise, N., Weih, F., et al. (2015). NEMO prevents steatohepatitis and hepatocellular carcinoma by inhibiting RIPK1 kinase activity-mediated hepatocyte apoptosis. Cancer Cell *28*, 582–598.

Legarda-Addison, D., Hase, H., O'Donnell, M.A., and Ting, A.T. (2009). NEMO/IKKgamma regulates an early NF-kappaB-independent cell-death checkpoint during TNF signaling. Cell Death Differ. *16*, 1279–1288.

Lewis, J., Devin, A., Miller, A., Lin, Y., Rodriguez, Y., Neckers, L., and Liu, Z.G. (2000). Disruption of hsp90 function results in degradation of the death domain kinase, receptor-interacting protein (RIP), and blockage of tumor necrosis factor-induced nuclear factor-kappaB activation. J. Biol. Chem. *275*, 10519–10526.

Li, H., Kobayashi, M., Blonska, M., You, Y., and Lin, X. (2006). Ubiquitination of RIP is required for tumor necrosis factor alpha-induced NF-kappaB activation. J. Biol. Chem. *281*, 13636–13643.

Mali, P., Yang, L., Esvelt, K.M., Aach, J., Guell, M., DiCarlo, J.E., Norville, J.E., and Church, G.M. (2013). RNA-guided human genome engineering via Cas9. Science *339*, 823–826.

Menon, M.B., Gropengießer, J., Fischer, J., Novikova, L., Deuretzbacher, A., Lafera, J., Schimmeck, H., Czymmeck, N., Ronkina, N., Kotlyarov, A., et al. (2017). $p38^{MAPK}$/MK2-dependent phosphorylation controls cytotoxic RIPK1 signalling in inflammation and infection. Nat. Cell Biol. *19*, 1248–1259.

Micheau, O., and Tschopp, J. (2003). Induction of TNF receptor I-mediated apoptosis via two sequential signaling complexes. Cell *114*, 181–190.

Micheau, O., Lens, S., Gaide, O., Alevizopoulos, K., and Tschopp, J. (2001). NF-kappaB signals induce the expression of c-FLIP. Mol. Cell. Biol. *21*, 5299–5305.

Moquin, D.M., McQuade, T., and Chan, F.K. (2013). CYLD deubiquitinates RIP1 in the TNFα-induced necrosome to facilitate kinase activation and programmed necrosis. PLoS ONE *8*, e76841.

O'Donnell, M.A., and Ting, A.T. (2011). RIP1 comes back to life as a cell death regulator in TNFR1 signaling. FEBS J. *278*, 877–887.

O'Donnell, M.A., Legarda-Addison, D., Skountzos, P., Yeh, W.C., and Ting, A.T. (2007). Ubiquitination of RIP1 regulates an NF-kappaB-independent cell-death switch in TNF signaling. Curr. Biol. *17*, 418–424.

Ofengeim, D., and Yuan, J. (2013). Regulation of RIP1 kinase signalling at the crossroads of inflammation and cell death. Nat. Rev. Mol. Cell Biol. *14*, 727–736.

Panayotova-Dimitrova, D., Feoktistova, M., Ploesser, M., Kellert, B., Hupe, M., Horn, S., Makarov, R., Jensen, F., Porubsky, S., Schmieder, A., et al. (2013). cFLIP regulates skin homeostasis and protects against TNF-induced keratinocyte apoptosis. Cell Rep. *5*, 397–408.

Ran, F.A., Hsu, P.D., Wright, J., Agarwala, V., Scott, D.A., and Zhang, F. (2013). Genome engineering using the CRISPR-Cas9 system. Nat. Protoc. *8*, 2281–2308.

Silke, J., and Meier, P. (2013). Inhibitor of apoptosis (IAP) proteins-modulators of cell death and inflammation. Cold Spring Harb. Perspect. Biol. *5*, 5.

Suda, J., Dara, L., Yang, L., Aghajan, M., Song, Y., Kaplowitz, N., and Liu, Z.X. (2016). Knockdown of RIPK1 markedly exacerbates murine immune-mediated liver injury through massive apoptosis of hepatocytes, independent of necroptosis and inhibition of NF-kappaB. J. Immunol. *197*, 3120–3129.

Tenev, T., Bianchi, K., Darding, M., Broemer, M., Langlais, C., Wallberg, F., Zachariou, A., Lopez, J., MacFarlane, M., Cain, K., and Meier, P. (2011). The Ripoptosome, a signaling platform that assembles in response to genotoxic stress and loss of IAPs. Mol. Cell *43*, 432–448.

Tokunaga, F., Sakata, S., Saeki, Y., Satomi, Y., Kirisako, T., Kamei, K., Nakagawa, T., Kato, M., Murata, S., Yamaoka, S., et al. (2009). Involvement of linear polyubiquitylation of NEMO in NF-kappaB activation. Nat. Cell Biol. *11*, 123–132.

Vanlangenakker, N., Bertrand, M.J., Bogaert, P., Vandenabeele, P., and Vanden Berghe, T. (2011). TNF-induced necroptosis in L929 cells is tightly regulated by multiple TNFR1 complex I and II members. Cell Death Dis. *2*, e230.

Varfolomeev, E., Blankenship, J.W., Wayson, S.M., Fedorova, A.V., Kayagaki, N., Garg, P., Zobel, K., Dynek, J.N., Elliott, L.O., Wallweber, H.J., et al. (2007). IAP antagonists induce autoubiquitination of c-IAPs, NF-kappaB activation, and TNFalpha-dependent apoptosis. Cell *131*, 669–681.

Vince, J.E., Wong, W.W., Khan, N., Feltham, R., Chau, D., Ahmed, A.U., Benetatos, C.A., Chunduru, S.K., Condon, S.M., McKinlay, M., et al. (2007). IAP antagonists target cIAP1 to induce TNFalpha-dependent apoptosis. Cell *131*, 682–693.

Wagner, S.A., Satpathy, S., Beli, P., and Choudhary, C. (2016). SPATA2 links CYLD to the TNF-α receptor signaling complex and modulates the receptor signaling outcomes. EMBO J. *35*, 1868–1884.

Walczak, H. (2011). TNF and ubiquitin at the crossroads of gene activation, cell death, inflammation, and cancer. Immunol. Rev. *244*, 9–28.

Walczak, H. (2013). Death receptor-ligand systems in cancer, cell death, and inflammation. Cold Spring Harb. Perspect. Biol. *5*, a008698.

Wang, L., Du, F., and Wang, X. (2008). TNF-alpha induces two distinct caspase-8 activation pathways. Cell *133*, 693–703.

Wong, W.W., Gentle, I.E., Nachbur, U., Anderton, H., Vaux, D.L., and Silke, J. (2010). RIPK1 is not essential for TNFR1-induced activation of NF-kappaB. Cell Death Differ. *17*, 482–487.

Zhang, J., Zhang, H., Li, J., Rosenberg, S., Zhang, E.C., Zhou, X., Qin, F., and Farabaugh, M. (2011). RIP1-mediated regulation of lymphocyte survival and death responses. Immunol. Res. *51*, 227–236.

Cell Reports, Volume *23*

Supplemental Information

Mind Bomb Regulates Cell Death during TNF Signaling by Suppressing RIPK1's Cytotoxic Potential

Rebecca Feltham, Kunzah Jamal, Tencho Tenev, Gianmaria Liccardi, Isabel Jaco, Celia Monteiro Domingues, Otto Morris, Sidonie Wicky John, Alessandro Annibaldi, Marcella Widya, Conor J. Kearney, Danielle Clancy, Paul R. Elliott, Timo Glatter, Qi Qiao, Andrew J. Thompson, Alexey Nesvizhskii, Alexander Schmidt, David Komander, Hao Wu, Seamus Martin, and Pascal Meier

Supplementary Information

Figure S1

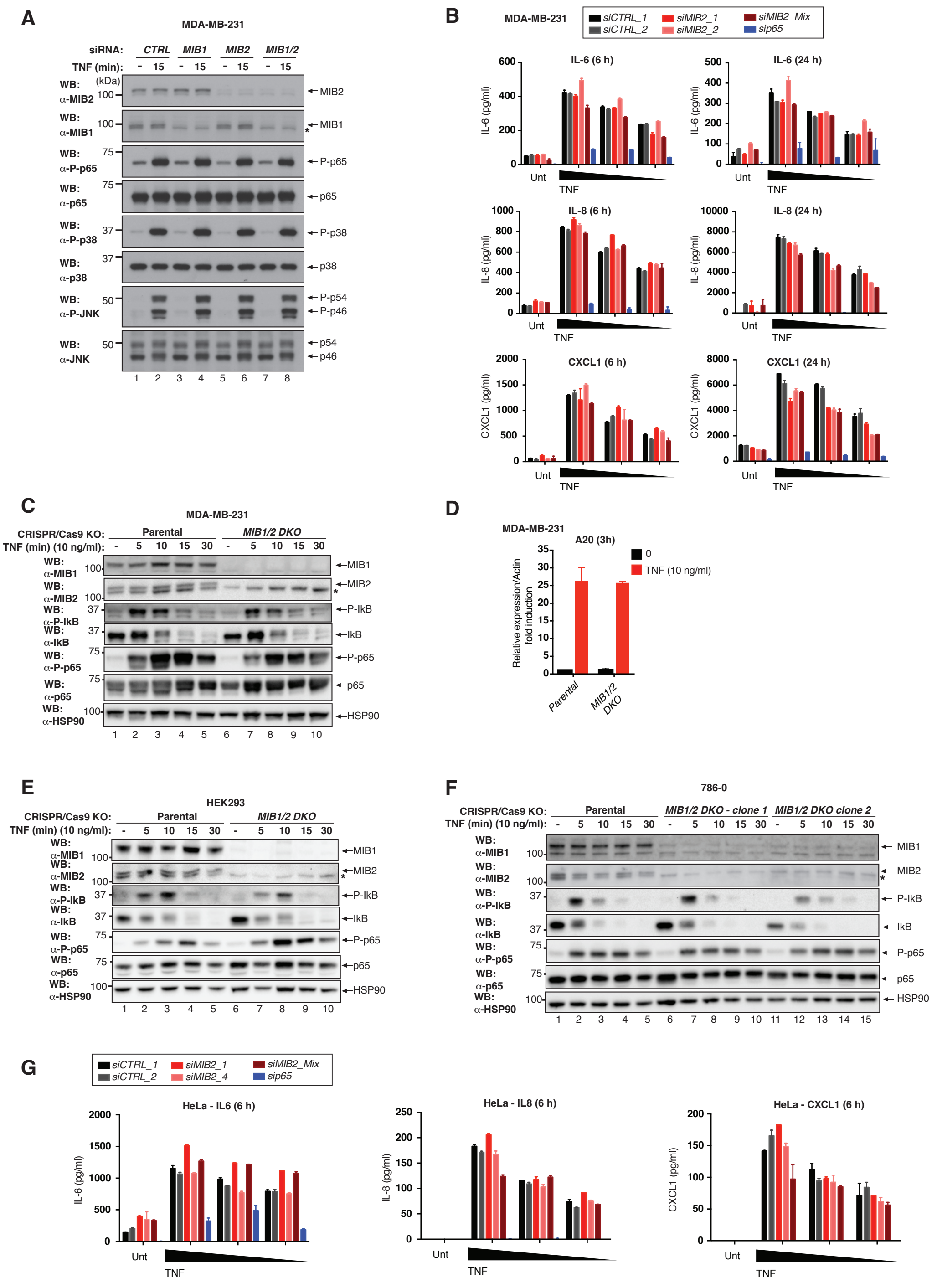

Supplementary Figure S1. MIB2 is not required for TNF-induced NF-ϰB activation and cytokine secretion, Related to Figure 1

(A) Western blot analysis of the indicated cell line subjected to RNAi-mediated knockdown of *MIB1*, *MIB2* or *MIB1/2*. Cells were either left untreated or treated with TNF (10 ng/ml) for 15 mins. (B) The presence of cytokines in the culture media of the indicated cell line was determined by ELISA. Cells were subjected to siRNA knockdown of *MIB2* or *p65* followed by stimulation with TNF at increasing concentrations (0.2-5 ng/ml) for 6 or 24 hrs. Error bars represent SD. (C) Comparison of TNF induced NF-ϰB activation in parental and *MIB1/2* DKO MDA-MB-231 cells. Cells were either left untreated or treated with TNF (10 ng/ml) for indicated times and lysates were analyzed by western blotting. (D) qRT-PCR analysis of mRNA from WT MDA-MB-231 and *MIB1/2* DKO MDA-MB-231 cells. Relative *A20* mRNA levels before and after 3 hrs of stimulation with 10 ng/ml TNF. (E-F) Comparison of TNF induced NF-ϰB activation in parental and *MIB1/2* DKO HEK293 cells (E) or 786-0 cells (F). Cells were either left untreated or treated with TNF (10 ng/ml) for indicated times and lysates were analyzed by western blotting. (G) The presence of cytokines in the culture media of HeLa cells was determined by ELISA. HeLa cells were subjected to siRNA-mediated knockdown of *MIB2* or *p65* followed by stimulation with TNF at increasing concentrations (0.2-5 ng/ml) for 6 or 24 hrs. Error bars represent SD.

Figure S2

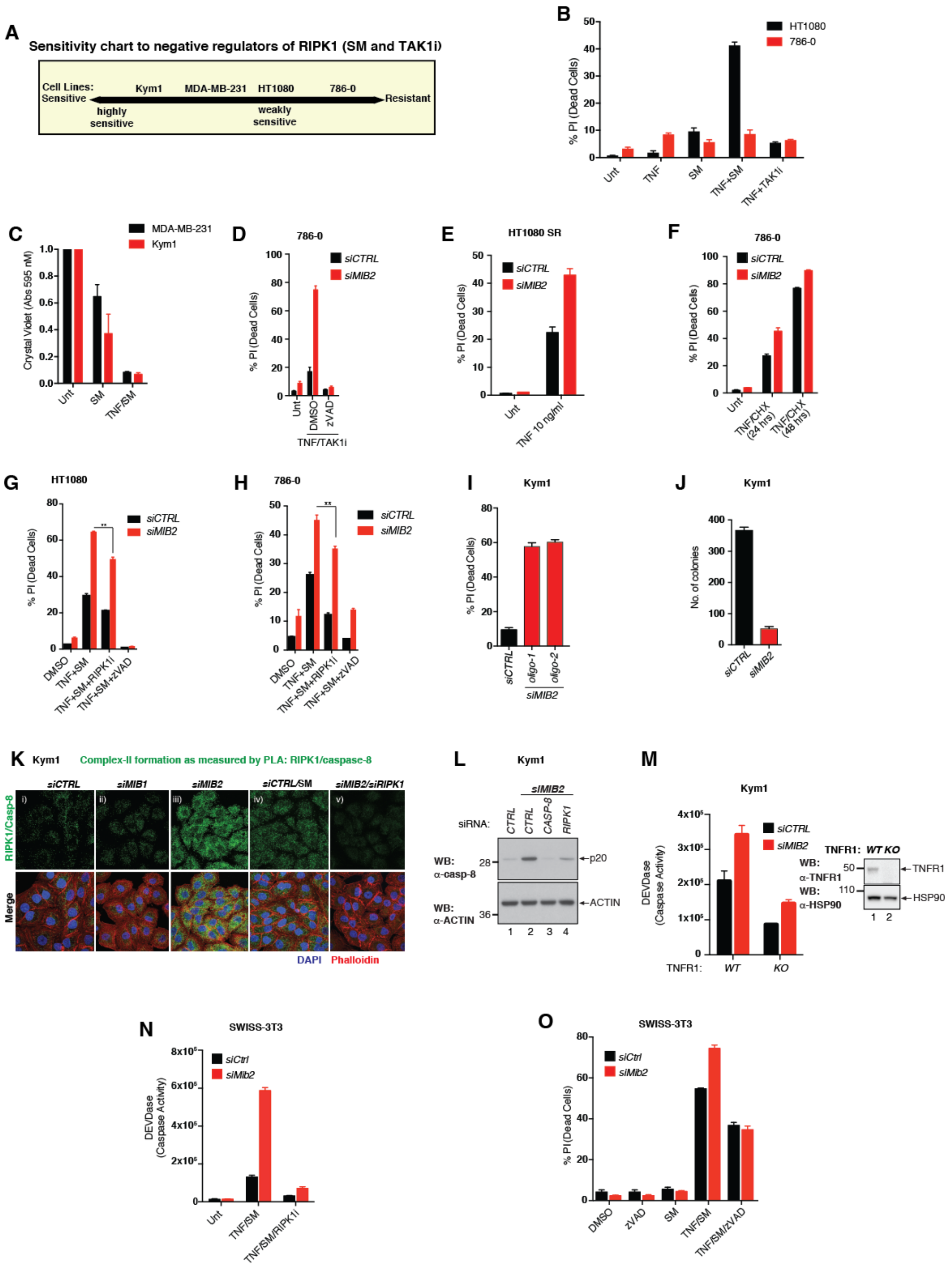

Supplementary Figure S2. MIB2 is required for TNF induced apoptosis, Related to Figure 2

(A) Schematic depicting the sensitivities of various cell lines to negative regulators of RIPK1. (B) FACS analysis of PI positive HT1080 and 786-0 cells treated with TNF (10 ng/ml), SM (100 nM), TNF/SM or TNF/TAK1i (1 μM) for 24 hrs. Error bars represent SD. (C) Crystal violet cell survival assay of Kym1 and MDA-MB-231 cells treated with SM (100 nM) or TNF (10 ng/ml)/SM (100 nM)

for 24 hrs. Error bars represent SD. (D) FACS analysis of PI positive 786-0 cells subjected to siRNA-mediated knockdown of *MIB2*. Following RNAi-mediated knockdown, cells were treated with DMSO or zVAD-FMK (10 μM) for 1 hr followed by treatment with TNF/TAK1i for 48 hrs. Error bars represent SD. (E) FACS analysis of PI positive HT1080 IkB^{SR} cells in which *MIB2* was knocked down by RNAi. Cells were treated with TNF (10ng/ml) for 24 hrs. Error bars represent SD. (F) FACS analysis of PI positive 786-0 cells subjected to siRNA-mediated knockdown of *MIB2*. Following RNAi-mediated knockdown, cells were treated with TNF (10 ng/ml) + CHX (10 μg/ml) for 24 or 48 hrs. Error bars represent SD. (G) Cell death analysis by Celigo of PI positive HT1080 cells subjected to siRNA-mediated knockdown of *MIB2*. Following RNAi-mediated knockdown, cells were treated with DMSO, zVAD-FMK (10 μM) or RIPK1i-GSK'963 (100 nM) for 1 hr followed by treatment with TNF/SM. Error bars represent SD. (H) Cell death analysis by Celigo of PI positive 786-0 cells subjected to siRNA-mediated knockdown of *MIB2*. Following RNAi-mediated knockdown, cells were treated with DMSO, zVAD-FMK (10 μM) or RIPK1i-GSK'963 (100 nM) for 1h followed by treatment with TNF/SM. Error bars represent SD. (I) FACS analysis of Kym1 cells treated with control siRNA oligos, or two independent oligos targeting *MIB2*. (J) Clonogenic growth assay using Kym1 cells subjected to siRNA knockdown of *MIB2*. 64 hrs post siRNA, 1000 cells were re-plated and left to form colonies. Error bars represent SEM. (K) Proximity ligation assay between RIPK1 and caspase-8 performed in Kym1 cells upon treatment with SM (100 nM) for 5 hrs or siRNA knockdown of *MIB1, MIB2, MIB2/RIPK1* for 96 hrs. All samples were treated with zVAD-FMK (10 μM) in fresh medium 16 hrs after transfection and then spiked with zVAD-FMK (10 μM) again at 48 hrs. (L) Western blot analysis of activated caspase-8 (p20 cleavage product) following siRNA knockdown of the indicated targets in Kym1 cells for 64 hrs. (M) DEVDase activity analysis of parental or *TNF-R1* KO Kym1 cells subjected to siRNA-mediated knockdown of *MIB2*. Following RNAi-mediated knockdown, cells were lysed and caspase activity was measured. (N) DEVDase assay using extracts from SWISS-3T3 cells subjected to siRNA knockdown of *Mib2*. Following RNAi-mediated knockdown, cells were treated with TNF/SM in presence or absence of RIPK1i-GSK'963 (100 nM) for 24 hrs. Error bars represent SD. (O) Cell death analysis by Celigo of PI positive SWISS-3T3 cells subjected to siRNA knockdown of *Mib2*. Following RNAi-mediated knockdown, cells were treated with the indicated agents for 17 hrs. Error bars represent SD.

Figure S3

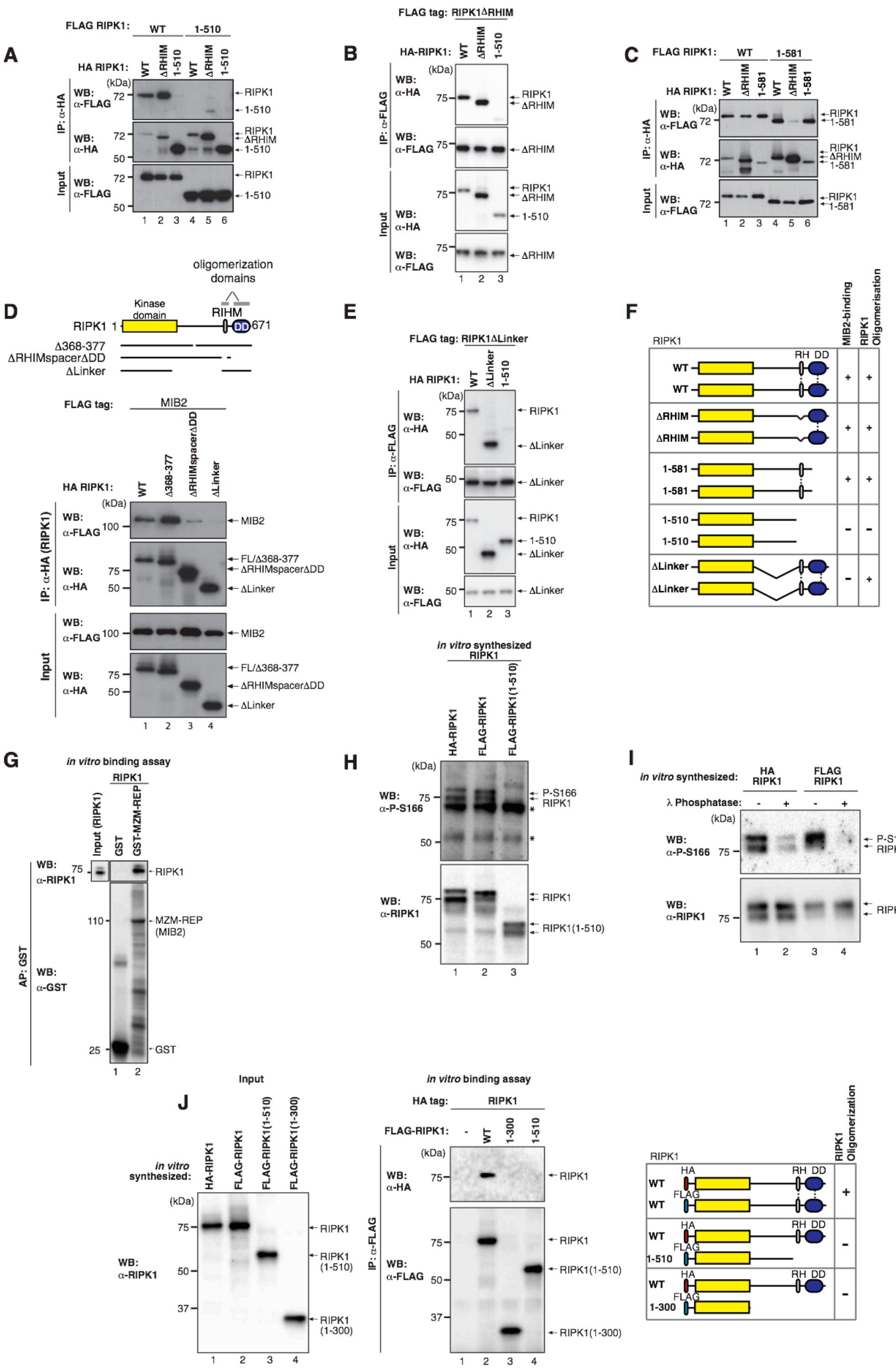

Supplementary Figure S3. MIB2 binds to oligomeric RIPK1, Related to Figure 3

(A-E) The indicated constructs were co-expressed in 293T cells. Immuno-precipitation was performed and interaction was assessed via western blot. (F) Schematic representation and summary of the results regarding MIB2 binding and RIPK1 homo-oligomerization. Indicated are the oligomerization and MIB2 binding capabilities of the various deletion constructs. (G) *In vitro* binding assay with

recombinant MIB2 and *in vitro* translated RIPK1. H) Western blot analysis of *in vitro* synthesized RIPK1 using the indicated antibodies. An asterisk indicates cross reactive bands. (I) Western blot analysis of *in vitro* synthesized RIPK1 treated with λ phosphatase as indicated. (J) *In vitro* binding assay of the indicated constructs. Left panel: input proteins. Middle panel: binding assay. FLAG-immuno-precipitation was performed and homo-oligomerization of *in vitro* synthesized RIPK1 proteins was assessed by Western blot. Right panel depicts the summary of the data.

Figure S4

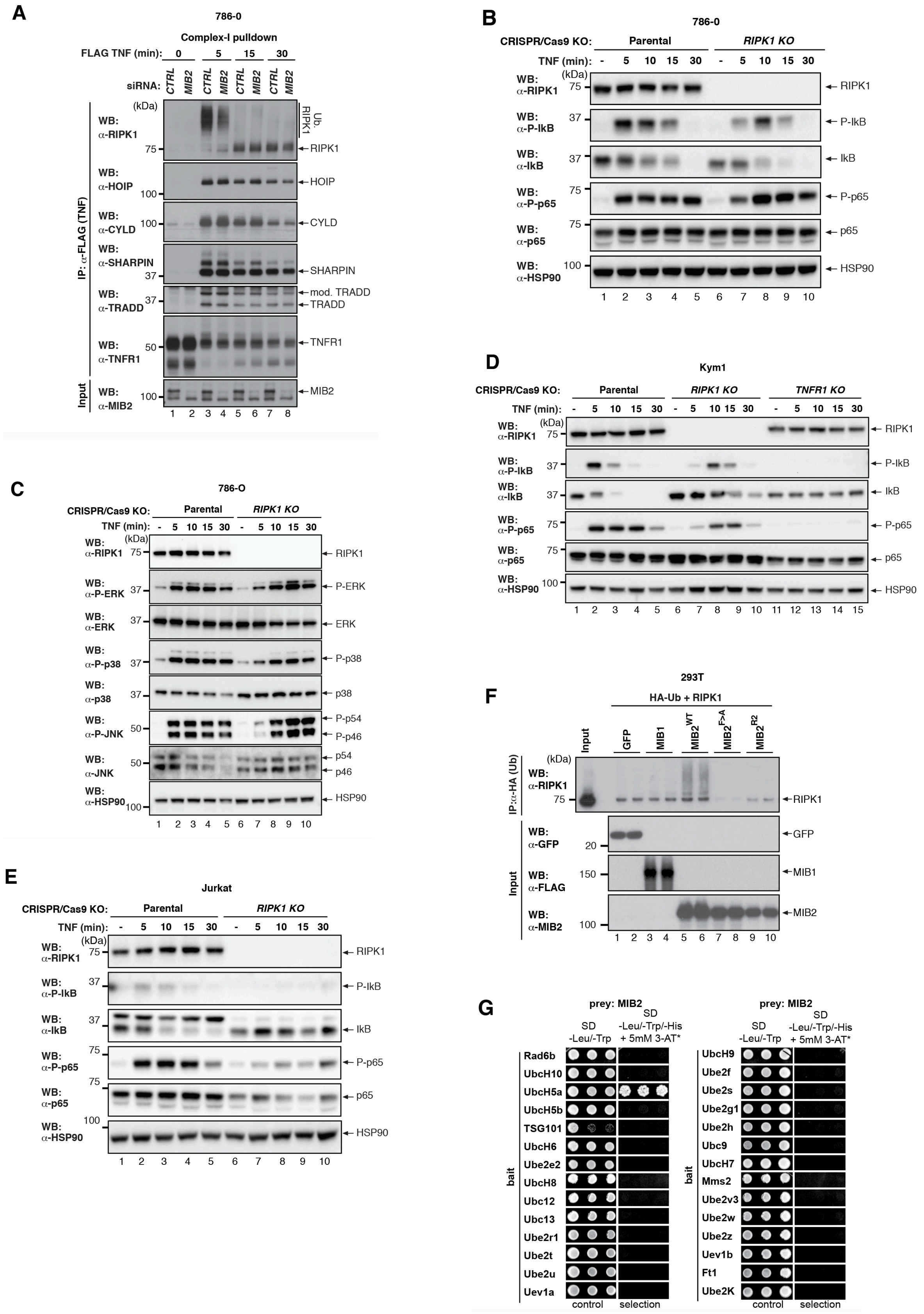
A
786-0
Complex-I pulldown
FLAG TNF (min): 0 5 15 30
siRNA: CTRL MIB2 CTRL MIB2 CTRL MIB2 CTRL MIB2
IP: α-FLAG (TNF)
Input
WB: α-RIPK1
WB: α-HOIP
WB: α-CYLD
WB: α-SHARPIN
WB: α-TRADD
WB: α-TNFR1
WB: α-MIB2
Ub. RIPK1
RIPK1
HOIP
CYLD
SHARPIN
mod. TRADD
TRADD
TNFR1
MIB2
B
786-0
CRISPR/Cas9 KO: Parental RIPK1 KO
TNF (min): - 5 10 15 30 - 5 10 15 30
WB: α-RIPK1
WB: α-P-IkB
WB: α-IkB
WB: α-P-p65
WB: α-p65
WB: α-HSP90
RIPK1
P-IkB
IkB
P-p65
p65
HSP90
C
786-O
CRISPR/Cas9 KO: Parental RIPK1 KO
WB: α-P-ERK
WB: α-ERK
WB: α-P-p38
WB: α-p38
WB: α-P-JNK
WB: α-JNK
P-ERK
ERK
P-p38
p38
P-p54
P-p46
p54
p46
D
Kym1
CRISPR/Cas9 KO: Parental RIPK1 KO TNFR1 KO
E
Jurkat
F
293T
HA-Ub + RIPK1
Input GFP MIB1 MIB2WT MIB2F>A MIB2R2
IP:α-HA (Ub)
WB: α-GFP
WB: α-FLAG
WB: α-MIB2
GFP
MIB1
MIB2
G
prey: MIB2
SD -Leu/-Trp
SD -Leu/-Trp/-His + 5mM 3-AT*
bait
Rad6b UbcH10 UbcH5a UbcH5b TSG101 UbcH6 Ube2e2 UbcH8 Ubc12 Ubc13 Ube2r1 Ube2t Ube2u Uev1a
UbcH9 Ube2f Ube2s Ube2g1 Ube2h Ubc9 UbcH7 Mms2 Ube2v3 Ube2w Ube2z Uev1b Ft1 Ube2K
control
selection

Supplementary Figure S4. RIPK1 is a substrate for MIB2, Related to Figure 5

(A) 786-0 cells were subjected to siRNA of *MIB2* followed by TNF-induced complex-I immuno-precipitation. Cells were treated with FLAG-hTNF (0.8□μg/ml) for 0, 5, 15 and 30 mins followed by FLAG immuno-precipitation and western blot analysis. (B) Comparison of TNF induced NF-ϰB activation in parental and *RIPK1* KO 786-0 cells. Cells were either left untreated or treated with TNF (10 ng/ml) for indicated times and lysates were analyzed by western blotting. (C) Comparison of TNF-induced activation of ERK, p38 and JNK in parental and *RIPK1* KO 786-O cells. Cells were either left untreated or treated with TNF (10 ng/ml) for indicated times and lysates were analyzed by western blotting. (D) Comparison of TNF induced NF-ϰB activation in parental, *RIPK1* KO and *TNF-R1* KO Kym1 cells. Cells were either left untreated or treated with TNF (10 ng/ml) for indicated times and lysates were analyzed by western blotting. (E) Comparison of TNF induced NF-ϰB activation in parental and *RIPK1* KO Jurkat cells. Cells were either left untreated or treated with TNF (10 ng/ml) for indicated times and lysates were analyzed by western blotting. (F) Untagged GFP, MIB1, $MIB2^{WT}$, $MIB2^{F>A}$ or $MIB2^{R2}$ was co-expressed with HA-Ub and untagged RIPK1 in 293T cells. HA-immuno-precipitation was performed and ubiquitylation of RIPK1 was assessed via western blot. (G) Yeast-two-hybrid assay screening the interaction of the RING finger of MIB2 (encoding amino acids 843-1000) with 22 human E2s and 6 human Ub-conjugating E2 variants (TSG101, Uev1a, Uev1b, Mms2, Ube2v3, Ft1).

Figure S5

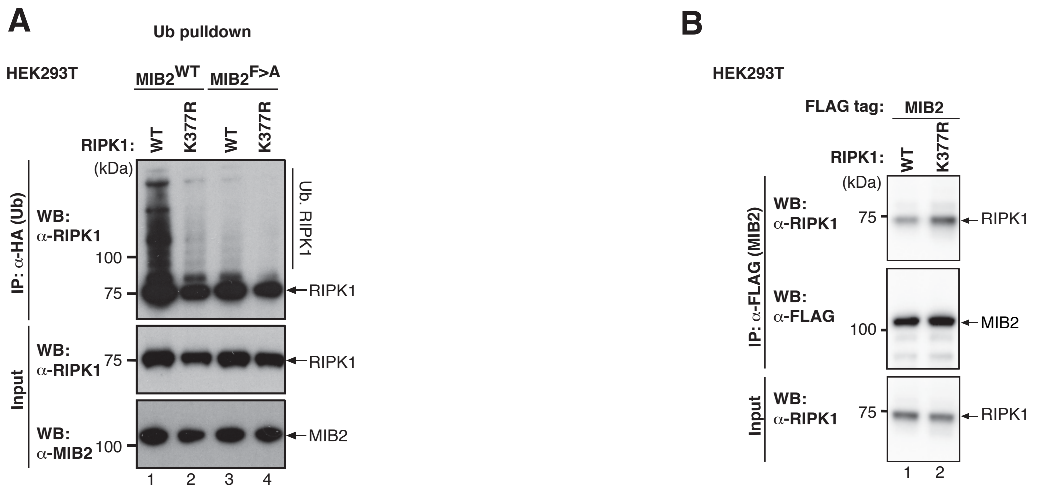

Supplementary Figure S5. RIPK1K377R mutation does not abrogate binding to MIB2, Related to Figure 6

(A) $RIPK^{WT}$ and $RIPK1^{K377R}$ were co-expressed with the indicated constructs in 293T cells. HA-immuno-precipitation was performed and ubiquitylation of RIPK1 was assessed via western blot. (B) FLAG MIB2 was co-expressed with untagged $RIPK1^{WT}$ or $RIPK1^{K377R}$ in 293T cells. FLAG-immuno-precipitation was performed and interaction with RIPK1 was assessed via western blot.

Figure S6

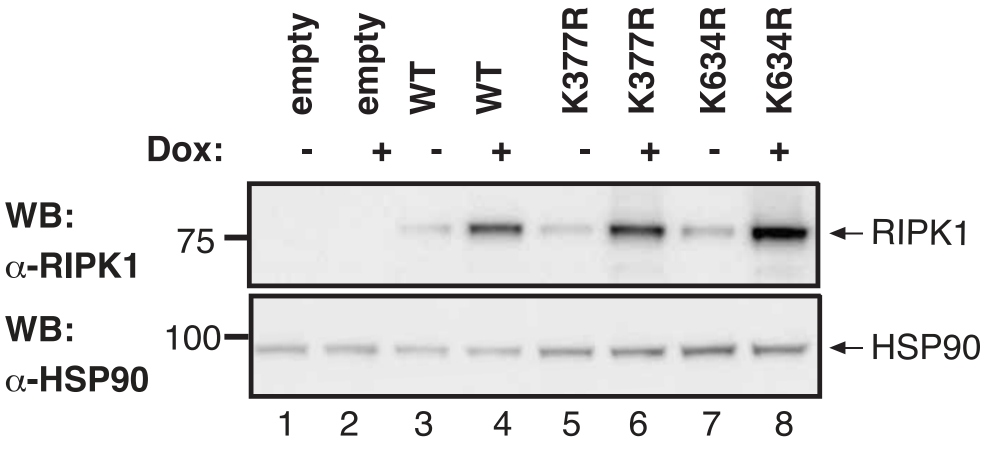

Supplementary Figure S6. Expression of RIPK1 mutants in MDA-MB-231 cells, Related to Figure 6

Western blot analysis of lysates from parental and *RIPK1* KO MDA-MB-231 cells reconstituted with either $RIPK1^{WT}$, $RIPK1^{K377R}$ and $RIPK1^{K377R}$ were induced with doxycycline for 6 hrs.

Figure S7

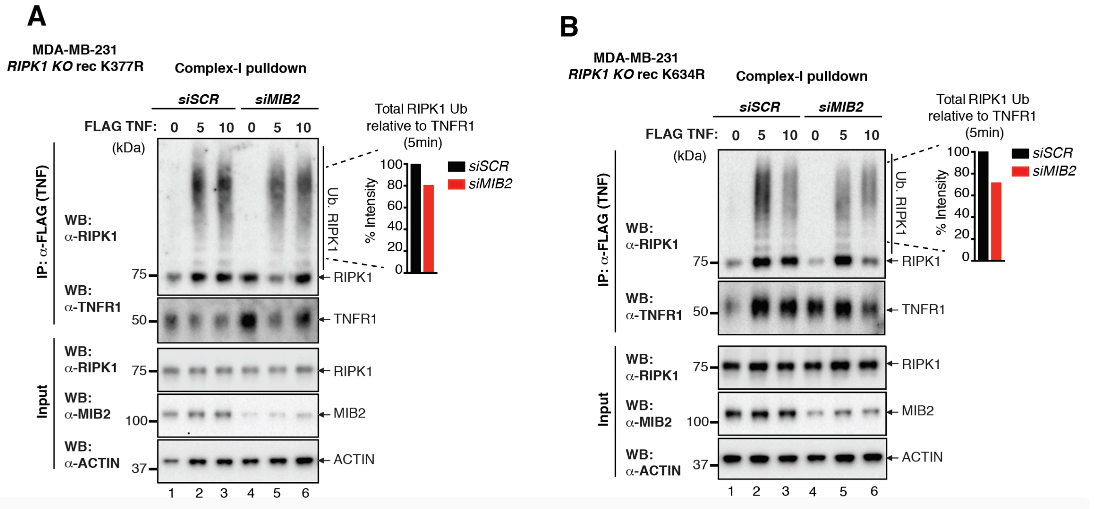

Supplementary Figure S7. MIB2 ubiquitylates RIPK1 at multiple lysine residues, Related to Figure 7

(A-B) Ubiquitylation of RIPK1 in complex-I. *RIPK1* KO MDA-MB-231 cells were reconstituted with either *RIPK1*^{WT} or *RIPK1*^{K377R} (A) or *RIPK1*^{K634R} (B). Cells were subjected to RNAi-mediated knockdown of *MIB2*. 48 hrs later cells were treated with FLAG-hTNF (0.8 μg/ml) for the indicated time-points post 5 hrs doxocycline induction, followed by FLAG immune-precipitation and western blot analysis.

Supplemental Experimental Procedures

Resource Table

REAGENT or RESOURCE	SOURCE	IDENTIFIER
Antibodies		
α-RIPK1 (N-terminal)	Cell Signaling	Cat#3493
α-HA	Roche	Cat#11867423001
α-MIB2	Bethyl Laboratories	Cat#A301-414A
α-MIB1	Gift from Patricia J. Gallagher	N/A
α-CYLD	Cell Signaling	Cat#8462; D1A10
α -SHARPIN	Proteintech	Cat#14626-1-AP
α-HOIL	Gift from Henning Walczak	N/A
α-HOIP	Bethyl Laboratories	Cat#A303-560A
α-NEMO	Santa Cruz Biotechnology	Cat#sc-8330
α-TRADD	BD Biosciences	Cat#610572
α-cIAP1	Enzo Life Sciences	Cat#ALX-803-335-C100
α-TAK1	Cell Signaling	Cat#4505
α-TNF-R1	Abcam	Cat#19139
α-TNF-R1	Santa Cruz Biotechnology	Cat#sc-8436
α-ACTIN	Sigma	Cat#A5441
α-P-p65	Cell Signaling	Cat#3033
α-p65	Cell Signaling	Cat#8242
α-IϰBα	Santa Cruz Biotechnology	Cat#sc-371
α-P-IϰBα	Cell Signaling	Cat#2859
α-P-p38	Cell Signaling	Cat#9215
α-p38	Cell Signaling	Cat#9212
α-P-JNK	Cell Signaling	Cat#4668
α-JNK	Santa Cruz Biotechnology	Cat#sc-571
α-P-ERK	Cell Signaling	Cat#9101
α-CASPASE-8 - for WB - post IP	MBL	Cat#M032-3
α-CASPASE-8 - for IP [C-20]	Santa Cruz Biotechnology	Cat#sc-6136
α-CASPASE-8 for cleavage	R&D	Cat#AF1650
α-FLAG [M2]	Sigma	Cat#F3165
α-Ub	Dako	Cat#Z0458
α-PARP1 [F2]	Santa Cruz Biotechnology	Cat#sc-8007
α-FLIP	Enzo Life sciences	Cat#ALX-804-428-C050
α-GFP	Santa Cruz Biotechnology	Cat#sc-8334
α-MYC	Sigma	Cat#M5546
α-HSP90	Santa Cruz Biotechnology	Cat#sc-7947
α-A20	Cell Signaling	Cat#5630
α-TRAF2	Santa Cruz Biotechnology	Cat#sc-876
α-ERK	Gift from Chris Marshall	N/A
α-pERK	Sigma	M8159
Chemicals, Peptides, and Recombinant Proteins		
Enbrel	Wyeth	N/A
Biotin-AHX-Ub-PA	(UbiQ)	Cat#UbiQ-076
FLAG-hTNF	Enzo Life Sciences	Cat#ALX-804-034-C050

FLAG-hTNF	Gift from Henning Walczak	N/A
(5Z)-7-Oxozeaenol (TAK1 inhibitor)	Tocris	Cat#3604
zVAD-FMK	Apex Bio	Cat#A1902
QVD	Apex Bio	Cat#A1901
SM-164	Gift from Shaomeng Wang	N/A
LPS	Invivogen	Cat#TLRL-PEKLPS
Ac-DEVD-AMC	Cambridge Bio	Cat#CAY14986
Hoechst	Thermo Scientific	Cat#33342
Propidium iodide solution (PI)	Sigma	Cat#P4864
MTT reagent	Sigma	Cat#M5655
Protein A/G agarose	Thermo Scientific	20423
Halt Protease and phosphatase inhibitor	Thermo Scientific	78443
PR619	2B Scientific	SI9619
GSK'963 (RIPK1 inhibitor)	Gift from GSK	N/A
Human TaqMan A20 Probe Hs00234713_m1	Thermo Scientific	Cat#4331182
Human TaqMan ACTIN Probe Hs01060665_g1	Thermo Scientific	Cat#4331182
Critical Commercial Assays		
RNAEasy	Qiagen	Cat#74106
QuantiTech reverse transcription	Qiagen	Cat#205314
Duolink In Situ Detection Reagents Green	Sigma	DUO92014
Experimental Models: Cell Lines		
HT1080^{IkB-SR}	Gift from O. Micheau (Dijon, France)	N/A
Kym1	Gift from John Silke (Melbourne, Australia)	N/A
HT1080	ATCC	Cat#CCL-121
MDA-MB-231	In house	N/A
HEK293T	In house	N/A
Flp-In™T-REx™-HEK293	Termo Scientific	Cat#R78007
786-0	In house	N/A
SWISS-3T3	In house	N/A
Oligonucleotides		
shRNA Mib2	Thermo Scientific	Clone ID: V3THS_324301
siMIB2_1 [Hs_ZZANK1_4 (hMib2)]	Qiagen	Cat#SI00779688
siMIB2_4 [hs_Mib2_4]	Qiagen	Cat#SI04369778
siMIB2_6 [hs_Mib2_6]	Qiagen	Cat#SI05126436
siALL*Control	Qiagen	Cat#1027281
Recombinant DNA		
Cas9-plasmid	Addgene	Cat#41815 or 48138
Mib2 cDNA	Gift from Vanessa Redecke	N/A
pcDNA3	Thermo Scientific	Cat#V79020
Deposited Data		
http://dx.doi.org/10.17632/52t6f2m8k5.1		
Software and Algorithms		

CRISPR design	http://crispr.mit.edu	(Ran et al., 2013)
CRISPR design	http://www.addgene.org/crispr/church/	(Mali et al., 2013)
SAINT analysis	http://saint-apms.sourceforge.net/	(Choi et al., 2011)
Swiss-Prot	https://www.ebi.ac.uk/uniprot	
Proteome Discoverer v1.4	Thermo Scientific	Cat#IQLAAEGABSFAKJMAUH
Image Lab V5.2.1.	Bio-Rad laboratories	
Sequence alignment	http://benchling.com	
GraphPad Prism v6.0	http://www.graphpad.com/	

CONTACT FOR REAGENTS AND RESOURCE SHARING

Further information and requests for reagents may be directed to Pascal Meier (pmeier@icr.ac.uk).

Plasmids

The *MIB2* cDNA (kind gift from Vanessa Redecke) was altered by mutagenesis to correspond to Q96AX9-2 - MIB2_human (UniProt). All constructs used for transient transfection experiments were cloned into pcDNA3 mammalian expression vector (Invitrogen) and sequence verified. For generation of stable cell lines Lentiviral tet-On inducible vector pTRB3A1 was used and cells were selected in the presence of Blasticidin.

RNA Interference, Transfections and Infections

Unless otherwise indicated, all siRNA assays were performed using a total of 50 nM – 100 nM of siRNA. When multiple siRNAs were combined, each siRNA was used at 25 nM and control siRNA was used where required to balance siRNA concentrations so equal amounts were transfected. All siRNA transfections unless otherwise stated were performed using DharmaFECT4 transfection reagent (GE Healthcare) and Opti-MEM (Life Technologies). All siRNA transfections unless otherwise stated were performed using retro transfection and left for 40 hrs from the time of transfection to facilitate knockdown. Unless otherwise indicated siMIB2 refers to the combination of hs_ZZANK1_4 (25 nM) + hs_MIB2_2 (25 nM) + hs_MIB2_4 (25 nM) + siCtrl (25 nM). For experiments where *siMIB1* and *siMIB2* are co-knocked down, *siMIB2* refers to the combination of hs_MIB2_2 (25 nM) + hs_MIB2_4 (25 nM) + *siCtrl* (50 nM) or *siMIB1* (50 nM). For all ELISA experiments $5x10^5$ cells were electroporated with 10 µl of 20 µM siRNA then seeded at $3x10^4$ in 24-well plates for 48 hrs. Generation of lentiviral particles was conducted as described previously (Vince et al., 2008; Vince et al., 2007).

Caspase-8 Cleavage Assays

Cells were seeded in 6-well plates and treated as indicated. Cells were lysed in 200 µl of DISC lysis buffer supplemented with 2% SDS, protease and phosphatase inhibitors. Cell lysates were passed through 0.8 ml columns (Pierce) to shred genomic DNA. Proteins lysates were quantified before separating samples by SDS-PAGE using NuPAGE Novex 4-12% Bis-Tris 1.0 mm 12 well precast

protein gels (Invitrogen) in MES buffer. Caspase-8 cleavage antibody [AF1650] (R&D) was used to detect cleavage products.

Cell Death (FACS) and MTT assays

Cells were plated in 96-well plates and retro siRNA transfection was performed for 40 hrs. Cells were treated as indicated in 150 µl for indicated times. Medium containing dead cells was transferred to a round bottom 96 well plate, live cells were trypsinized in 50 µl, live cells were harvested with 100 µl of medium containing 1 µg/ml PI with 2.5 mM $CaCl_2$ and 2.5 µl/ml AnnexinV antibody (BD Biosciences) and combined with dead cells (total volume 300 µl). 96-well plate was analyzed by FACS using a plate reader. Data shown are from 5000 cells per condition. For CeligoS assays, 5 x10^4 786-O or HT1080 cells were seeded in 96-well plates and 24 h later cells were treated as indicated for the indicated times. Hoechst (0.5 µg/ml) and PI (1 µg/ml) were added and the percentage of dead cells was measured using the CeligoS image cytometer (Nexcelon Bioscience). For MTT assays 5x10^4 cells were seeded in 24-well plates. Cells were treated as indicated in 500 µl for indicated times, after which 50 µl of MTT reagent re-suspended in $H_2$0 to a concentration of 5 mg/ml was added to the cells and left to develop for 2 hrs. Media was removed and 500 µl DMSO added to solubilize the crystals. Absorbance was read on a spectrometer at 570 nm.

Crystal Violet Cell Survival Assays

Cells were seeded in a 6-well plate. The following day the indicated treatments were added in 2 ml of DMEM and the cells were left for 24 hrs in treated medium. Cells were fixed in 3.7% formaldehyde/PBS for 10 mins and stained with crystal violet/PBS for 10 mins. Colonies were either dissolved in 1 ml of 10% acetic acid and the absorbance read on a spectrometer at 595 nm.

Clonogenic Assays

Cells were seeded in a 6-well plate and retro siRNA transfection was performed. After 40 hrs (786-0) or 64 hrs (Kym1) cells were trypsinized, counted and 1000 viable cells were re-plated into a 6-well plate. The following day the indicated treatments were added in 2 ml of DMEM and the cells were left for approximately 9 days in treated medium. Cells were fixed in 3.7 % formaldehyde/PBS for 10 mins and stained with crystal violet/PBS for 10 mins. Colonies were either dissolved in 1 ml of 10% acetic acid and the absorbance read on a spectrometer at 595 nm, or the colonies were counted and recorded using Image J.

Caspase activity assays (DEVDase)

DEVDase assay was performed as previously described (Jaco et al., 2017). In brief, cells were plated in 96-well plates and retro siRNA transfection was performed for 40 hrs. After treatment, medium was removed and 1 % DISC lysis buffer (20 mM Tris-HCL pH7.5, 150 mM NaCl, 2 mM EDTA, 1 % Triton X-100, 10 % Glycerol, $H_2$0) was added to each well. Plates were placed at -80 °C to aid cell lysis. Plates were thawed at room temperature for 15 mins, after which DEVDase assay mix was added to each well (NB: cell lysates were not cleared). The plates were wrapped in foil and the reaction was

incubated at room temperature for up to 24 hrs. DEVDase activity was read at 380 nM excitation/460 nM emission.

TUBE Assays

Cells were lysed in DISC lysis buffer (20 mM Tris-HCL pH7.5, 150 mM NaCl, 2mM EDTA, 1% Triton X-100, 10% Glycerol, H_20) supplemented with protease inhibitors, 1 mM DTT, PR619 (10 µM), GST-TUBE (50 µg/ml; 50 µg TUBE/mg protein lysate). Cell lysates were rotated at 4 °C for 20 mins then clarified at 4 °C at 14,000 rpm for 10 mins. 20 µl GST beads were added and immuno-precipitation was performed overnight. 4x washes in wash buffer (50 mM Tris pH 7.5, 150 mM NaCl, 0.1 % Triton X-100, and 5 % glycerol) + PR619 (10 µM) were performed, and samples eluted by boiling in 50 µl 1x SDS loading dye.

Homology Modeling

A homology model of human RIPK1 Death Domain (DD, 583-669) was generated by the SWISS-MODEL server using PIDD DD structure as the template (PDB code 2OF5). Then modeled RIPK1 DD structure was aligned to FAS DD in FAS/FADD complex structure (PDB code 3OQ9) to form a RIPK1 DD/FADD DD complex structure. The alignment was performed by Coot and figures were made by PyMol.

Complex-I/II Purification

Complex-I/II purification was essentially performed as previously described (Jaco et al., 2017). In brief, cells were seeded in 15 cm dishes and treated as indicated using pre-warmed media containing 3xFLAG-hTNF (0.8 µg/ml). After stimulation media was removed and plates were washed with ice cold PBS to stop stimulation and frozen at -80 °C. Plates were thawed and cells were lysed in DISC lysis buffer supplemented with protease inhibitors and PR619 (10 µM). Cell lysates were rotated at 4 °C for 20 mins then clarified at 4 °C at 14,000 rpm for 10 mins. 20 µl of anti-FLAG M2 beads (SIGMA) were rotated with cleared protein lysates overnight at 4 °C. 0 hr sample: 0.8 µg/ml of FLAG-TNF was added post-lysis. 4x washes in DISC buffer with PR619 (10 µM) were performed, and samples eluted by boiling in 50 µl 1x SDS loading dye. For complex-II purification cells were seeded in 10 cm dishes and treated as indicated in figure legends. Cells were lysed on ice as above. Cell lysates were rotated at 4 °C for 20 mins then clarified at 4 °C at 14,000 rpm for 10 mins. 20 µl of protein G sepharose (SIGMA) with Caspase-8 (C20) antibody (Santa Cruz Biotechnology) (1.5 µg antibody/mg protein lysate) were rotated with cleared protein lysates overnight at 4 °C. 4x washes in wash buffer (50 mM Tris pH 7.5, 150 mM NaCl, 0.1% Triton X-100, and 5% glycerol) were performed, and samples eluted by boiling in 50 µl 1x SDS loading dye.

Ubiquitylation Assays

0.4 µg of plasmids expressing HA-Ub or Myc-Ub was transfected into 293T cells in combination with the indicated constructs. Transfection was left for 16 hrs after which cells were lysed with DISC lysis buffer supplemented with protease inhibitors and PR619 (10 µM). Cell lysates were rotated at 4 °C for

20 mins then clarified at 4°C at 14,000 rpm for 10 mins. 20 µl anti-HA beads (SIGMA) or 20 µl of protein G sepharose (SIGMA) + 3 µl anti-Myc were rotated with cleared protein lysates overnight at 4 °C. 4x washes in wash buffer (50 mM Tris pH 7.5, 150 mM NaCl, 0.1% Triton X-100, and 5% glycerol) supplemented with PR619 (10 µM) were performed, and samples eluted by boiling in 50 µl 1x SDS loading dye.

In vitro Binding and Ubiquitylation Assay

All constructs for *in vitro* translation assays were cloned into pcDNA3 and translated using the Promega TNT Coupled Reticulocyte Lysate System. For production of GST-tagged proteins, MIB2 constructs were expressed from pGEX6p-1 in BL21(DE3)/pLysS strain and purified with GST beads. All *in vitro* binding assays were conducted in the presence of DISC buffer overnight at 4 °C. Beads were washed 4 times with the same buffer and protein complexes were eluted by boiling the beads in 1x SDS loading dye. For the *in vitro* assay shown in Fig 5G, purified Strep-tagged MIB2 was incubated with recombinant GST-tagged full length RIPK1 (Abnova), E1 enzyme preloaded with Ub (Boston Biochem), UbcH5a (Boston Biochem) and ATP in Ub assay buffer (40 mM Tris-HCL pH 7.5, 10 mM MgCl2, 0.6 mM DTT) at 37 °C for 90 mins. Reactions were stopped by adding SDS loading dye and samples were analysed by Western blot with the indicated antibodies.

UbiCRest

The UbiCRest analysis with linkage selective DUBs was performed essentially as previously described (Hospenthal et al., 2015). Briefly, the release fraction (see above) was incubated with the following DUBs: 1 µM OTULIN, 0.2 µM OTUD1, 1 µM CEZANNE, 0.2 µM OTUB1, 1.5 µM USP21, 0.5 µM vOTU. The reaction was conducted in the presence of 1 mM DTT for 30 min at 37 °C. Reactions were stopped with loading buffer, and the ubiquitylation status analyzed by western blotting.

Proximity Ligation Assay

PLA was performed according to the manufacturer's protocol using the Duolink Detection Kit (SIGMA). Cells were examined with a confocal microscope (objective x 40, Zeiss LSM 710).

Directed Yeast Two-Hybrid Assays

The yeast strain Y2HGold (Clontech) was co-transformed with pGBT9-MIB2 (encoding amino acids 843-1000) as a bait and the respective prey plasmids encoding 22 human E2s and 6 human Ub-conjugating E2 variants. Positive transformants were selected on minimal SD-Leu-Trp medium (Formedium). Three single colonies for each bait and prey co-transformation were patched out on fresh SD-Leu-Trp plates and grown for 2 days at 30 °C. Each patch was re-suspended in 180 µl of sterile water in a 96 well plate and plated in replicate onto non-selective (SD-Leu-Trp) or selective medium (SD-Leu-Trp-His, containing 5 mM of 3-amino-1,2,4-triazole (3-AT, Formedium)). Yeasts were incubated at 30ºC for 1 week. The E2s/UEVs library was kindly provided by Rachel Klevit.

qRT-PCR

qRT-PCR was performed as previously described (Morris et al., 2016), with some modifications. MDA-MB-231 parental and *MIB1/2 DKO* were treated with TNF (10 ng/ml) for 3 hrs and immediately frozen. qRT-PCR was performed using Taqman gene expression mastermix (Thermo Fisher Scientific) and the QuantStudio 6 Flex Real-Time PCR System. The amount of mRNA detected was normalized to control *ACTIN* mRNA values. The relative ΔCt sample/ΔCtr Actin ratios of WT controls were set at 100%, and the fold differences were calculated using the ΔΔCt method.

Mass Spectrometry

Prior to mass spectrometry analysis of RIPK1 interactors, eluted protein complexes were digested with Trypsin and peptides were purified using C18 Microspin columns (Harvard Apparatus) according to the manufactures instruction. LC-MS/MS analysis was performed on a dual pressure LTQ-Orbitrap mass spectrometer (Thermo Scientific), which was connected to an electrospray ion source (Thermo Scientific). Peptide separation was carried out using an easy nano-LC systems (Proxeon Biosystems) equipped with an RP-HPLC column packed with C18 resin (Magic C18 AQ 3 μm; Michrom BioResources). A 0.3 μl/min linear gradient from 96 % solvent A (0.15 % formic acid, 2 % acetonitrile) and 4 % solvent B (98 % acetonitrile, 0.15 % formic acid) to 40 % solvent B over 40 min. The data acquisition mode was set to obtain one high-resolution MS scan in the FT part of the mass spectrometer at a resolution of 60,000 FWHM followed by MS/MS scans in the linear ion trap of the 20 most intense ions. Raw files were converted to the mzXML format, and searched against the human swissprot protein database. Further data processing including SAINT was carried out as described previously (Choi et al., 2011). For the identification of ubiquitylated sites on RIPK1 by MIB2, 293T cells were transfected with 3xHA-RIPK1, MIB2 and Ub or 3xHA-RIPK1, MIB2F>A and Ub for 48 hrs. After lysis with DISC lysis buffer in the presence of protease inhibitors and PR619, HA affinity purification was performed. Bound complexes were eluted with 5 % formic acid and then submitted for analysis by LC-MS/MS using a tryptic digestion workflow. Specific accurate mass (+/- 10 ppm) and retention time (AMRT) profiles were determined for the peptides of interest, accounting for the following variable modification states: unmodified and diGly-modification of lysine. Additionally, the sum value of four RIPK1 peptides was calculated relative to the data from the RIPK1 sample. The four peptides were selected as proxies to indicate the level of RIPK1 in the two samples. LC-MS/MS analysis of ubiquitylated peptides was performed after immuno-precipitation. Elutes were dried in vacuo and reconstituted in 50 mM triethylammonium bicarbonate. Samples were then reduced with 5 mM tris(2-carboxyethyl)phosphine, free cysteines were alkylated with 10 mM chloroacetamide or chloroacetic acid and protein was digested with trypsin. For the targeted analysis of residue K377, a second digestion was performed using endoproteinase Glu-C. The resulting peptides were analysed by direct injection on an Agilent 1200 nanoLC (Agilent Technologies) in-line with an LTQ Velos Orbitrap mass spectrometer (Thermofisher Scientific) with the following modifications: Peptides were resolved over 30 mins using a linear gradient of 96:4 to 50:50 buffer A:B (buffer A: 1% acetonitrile/3 % dimethyl sulfoxide/0.1 % formic acid; buffer B: 80 % acetonitrile/3 % dimethyl sulfoxide/0.1 % formic acid) at 250 nL/min. The ion at 401.922718 m/z was used for FT-MS internal lock mass calibration. Peak lists were extracted using Proteome Discoverer v1.4 and interrogated using Mascot v2.3 against

the Swissprot 2015_04 Homo Sapiens subset database (20,273 sequences) customized to include construct sequences as required. Residual Ub signatures GG and LRGG were included as variable modifications at lysine residues.

Supplemental References

Choi, H., Larsen, B., Lin, Z.Y., Breitkreutz, A., Mellacheruvu, D., Fermin, D., Qin, Z.S., Tyers, M., Gingras, A.C., and Nesvizhskii, A.I. (2011). SAINT: probabilistic scoring of affinity purification-mass spectrometry data. Nat Methods *8*, 70-73.

Hospenthal, M.K., Mevissen, T.E., and Komander, D. (2015). Deubiquitinase-based analysis of ubiquitin chain architecture using Ubiquitin Chain Restriction (UbiCRest). Nature protocols *10*, 349-361.

Jaco, I., Annibaldi, A., Lalaoui, N., Wilson, R., Tenev, T., Laurien, L., Kim, C., Jamal, K., Wicky John, S., Liccardi, G., *et al*. (2017). MK2 Phosphorylates RIPK1 to Prevent TNF-Induced Cell Death. Mol Cell *66*, 698-710 e695.

Mali, P., Yang, L., Esvelt, K.M., Aach, J., Guell, M., DiCarlo, J.E., Norville, J.E., and Church, G.M. (2013). RNA-guided human genome engineering via Cas9. Science *339*, 823-826.

Morris, O., Liu, X., Domingues, C., Runchel, C., Chai, A., Basith, S., Tenev, T., Chen, H., Choi, S., Pennetta, G., *et al*. (2016). Signal Integration by the IkappaB Protein Pickle Shapes Drosophila Innate Host Defense. Cell Host Microbe *20*, 283-295.

Vince, J.E., Chau, D., Callus, B., Wong, W.W., Hawkins, C.J., Schneider, P., McKinlay, M., Benetatos, C.A., Condon, S.M., Chunduru, S.K., *et al*. (2008). TWEAK-FN14 signaling induces lysosomal degradation of a cIAP1-TRAF2 complex to sensitize tumor cells to TNFalpha. J Cell Biol *182*, 171-184.

Vince, J.E., Wong, W.W., Khan, N., Feltham, R., Chau, D., Ahmed, A.U., Benetatos, C.A., Chunduru, S.K., Condon, S.M., McKinlay, M., *et al*. (2007). IAP antagonists target cIAP1 to induce TNFalpha-dependent apoptosis. Cell *131*, 682-693.
